# Circadian clock component PERIOD2 regulates diurnal expression of Na^+^/H^+^ exchanger regulatory factor-1 and its scaffolding function

**DOI:** 10.1038/s41598-018-27280-w

**Published:** 2018-06-13

**Authors:** Yuya Tsurudome, Satoru Koyanagi, Takumi Kanemitsu, Chiharu Katamune, Masayuki Oda, Yuki Kanado, Mizuki Kato, Akari Morita, Yu Tahara, Naoya Matsunaga, Shigenobu Shibata, Shigehiro Ohdo

**Affiliations:** 1 Department of Pharmaceutics, Faculty of Pharmaceutical Sciences, Kyushu University, Fukuoka, Japan; 2 Department of Glocal Healthcare Science, Faculty of Pharmaceutical Sciences, Kyushu University, Fukuoka, Japan; 3 Department of Psychiatry and Behavioral Sciences, University of California, Los Angeles, CA 90024 USA; 4 Laboratory of Physiology and Pharmacology, School of Advanced Science and Engineering, Waseda University, Shinjuku-ku, Tokyo, Japan

## Abstract

A number of diverse cell-surface proteins are anchored to the cytoskeleton via scaffold proteins. Na^+^/H^+^ exchanger regulatory factor-1 (NHERF1), encoded by the *Slc9a3r1* gene, functions as a scaffold protein, which is implicated in the regulation of membrane expression of various cell-surface proteins. Here, we demonstrate that the circadian clock component PERIOD2 (PER2) modulates transcription of the mouse *Slc9a3r1* gene, generating diurnal accumulation of NHERF1 in the mouse liver. Basal expression of *Slc9a3r1* was dependent on transcriptional activation by p65/p50. PER2 bound to p65 protein and prevented p65/p50-mediated transactivation of *Slc9a3r1*. The time-dependent interaction between PER2 and p65 underlay diurnal oscillation in the hepatic expression of *Slc9a3r1*/NHERF1. The results of immunoprecipitation experiments and liquid chromatography-mass spectrometry analysis of mouse liver revealed that NHERF1 time-dependently interacted with fatty acid transport protein-5 (FATP5). Temporary accumulation of NHERF1 protein stabilized plasmalemmal localization of FATP5, thereby enhancing hepatic uptake of fatty acids at certain times of the day. Our results suggest an unacknowledged role for PER2 in regulating the diurnal expression of NHERF1 in mouse liver. This machinery also contributed to diurnal changes in the ability of hepatic cells to uptake fatty acids.

## Introduction

A number of biological and physiological processes are subjected to diurnal regulation. Time-dependent variations are driven by an endogenous time-keeping system called the circadian clock. The core circadian oscillator constitutes transcription-translation feedback loops. A heterodimer consisting of CLOCK and BMAL1 enhances the transcription of *Period* (*Per*) and *Cryptochrome* (*Cry*) genes by binding to E-box elements^1–3^. Once PER and CRY proteins reach critical concentrations, they attenuate CLOCK/BMAL1-mediated transactivation, thereby generating circadian oscillations in their own transcription. The transcription of REV-ERBα is also activated by CLOCK/BMAL1, and its transactivation is repressed by PER and CRY proteins, resulting in circadian oscillations in the expression of REV-ERBα encoded by the *Nr1d1* gene. REV-ERBα, in turn, periodically represses retinoic acid receptor-related orphan receptor-α (RORα)-mediated *Bmal1* transcription through orphan receptor response elements (ROREs), thereby interconnecting positive and negative feedback loops^4,5^. Similar to the mechanism underlying *Nr1d1* transcription, clock genes consisting of core oscillation loops control the circadian expression of clock-controlled output genes, such as albumin D-site binding protein (DBP), E4BP4 (also called as NFIL3), and peroxisome proliferator-activated receptor-α (PPARα)^6–8^. DBP and E4BP4 regulate circadian gene expression by competitively binding to the same DNA sequence D-site^6,7^. Similarly, PPAR response elements (PPREs) are required for the circadian expression of PPARα-target genes^8^. These mechanisms ultimately control various downstream events in the transcription, translation, and degradation processes.

Cell-surface proteins, such as receptors, channels, and transporters are crucial for signal transduction elicited by extracellular stimuli, the uptake of nutrients, and/or the efflux of waste. The expression of these membrane proteins is often anchored by scaffold proteins^9,10^. Proteins containing PDZ domains (PSD95, DLG1, and ZO1), which are critical protein-protein recognition motifs, play important roles in anchoring cell-surface proteins to cytoskeletal components^11,12^. Consequently, scaffold proteins constitute the fundamental building blocks of membrane-protein complexes^13^. Na^+^/H^+^ exchanger regulatory factor-1 (NHERF1), encoded by the *Slc9a3r1* gene, has been implicated in the regulation of membrane retention, endocytic sorting, and epithelial morphogenesis^14,15^. NHERF1 also contains PDZ domains and, thus, acts as a scaffold protein^16,17^. Diurnal variations in *Slc9a3r1* mRNA levels have been detected in the rat colonic mucosa^18^; however, the mechanisms underlying the diurnal expression of *Slc9a3r1*/NHERF1 and its physiological significance remain unknown.

In the present study, we found that the expression of *Slc9a3r1*/NHERF1 was controlled by the molecular component of the circadian clock PER2. This circadian transcriptional repressor time-dependently suppressed the p65/p50-mediated transactivation of *Slc9a3r1*, thereby generating diurnal accumulation of NHERF1 protein. The results of immunoprecipitation experiments and liquid chromatography-mass spectrometry analysis revealed that NHERF1 time-dependently interacted with fatty acid transporter protein-5 (FATP5). Periodic accumulation of the NHERF1 protein caused diurnal changes in the localization FATP5 on hepatic plasma membrane. Our results suggest a previously unacknowledged role of PER2 in regulating the diurnal expression of NHERF1, contributing to diurnal changes in hepatic fatty acid uptake.

## Results

### PER2 represses p65/p50-mediated transactivation of *Slc9a3r1*

**Figure 1:**
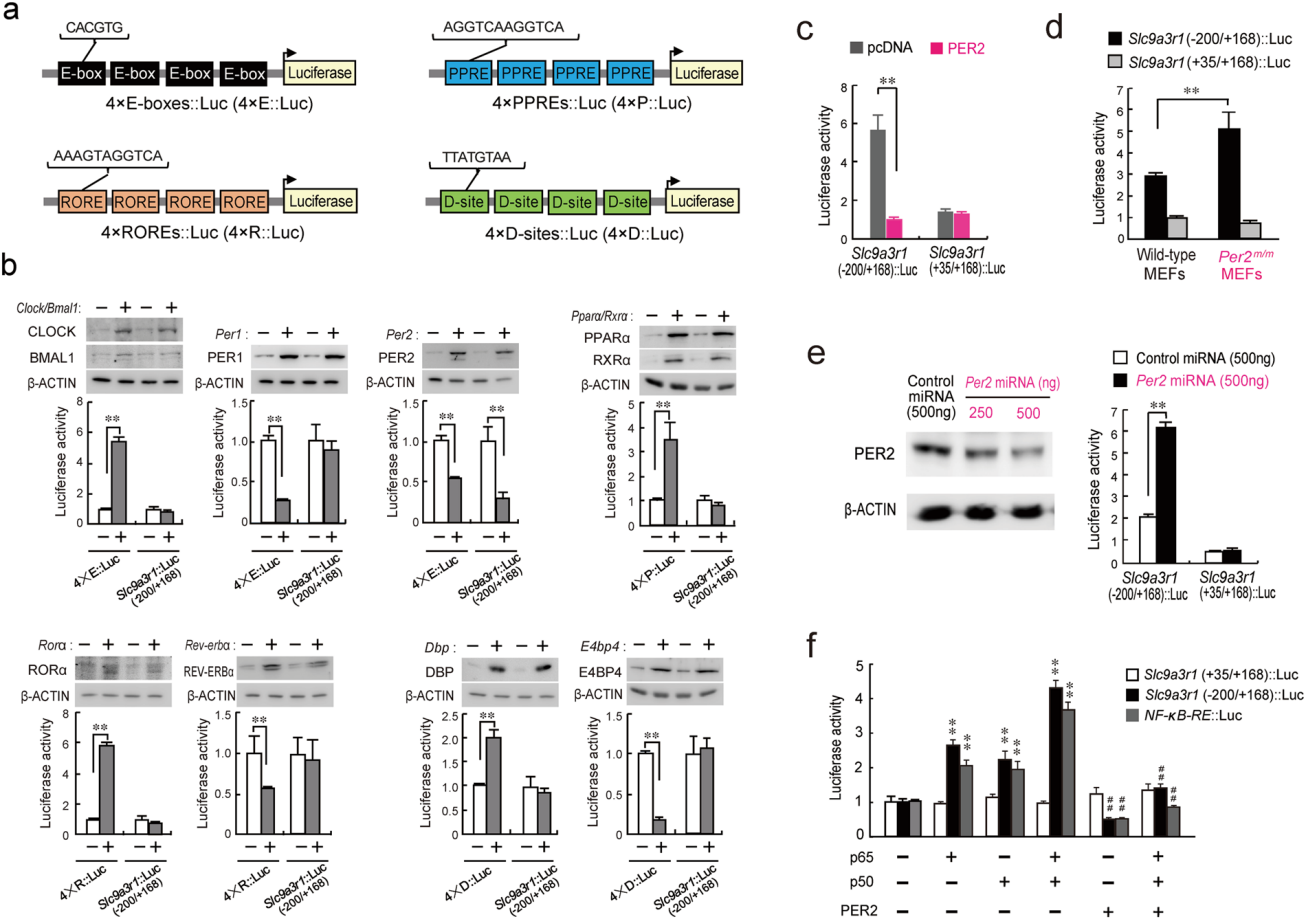
PER2 represses p65/p50-mediated *Slc9a3r1* transcription. (**a**) Schematic representation of luciferase reporter constructs that contain tandemly repeated E-boxes (4 × E-boxes::Luc), ROREs (4 × ROREs::Luc), D-sites (4 × D-sites::Luc), or PPREs (4 × PPREs::Luc). (**b**) Effects of clock gene products on the transcriptional activities of 4 × E-boxes::Luc, 4 × ROREs::Luc, 4 × D-sites::Luc, 4 × PPREs::Luc, and *Slc9a3r1*(−200/+168)::Luc. Control groups were transfected with empty vector (pcDNA3.1) instead of expression plasmids. Presence (+) or absence (−) of expression plasmids (0.5 µg each) is denoted. Each value represents the mean ± s.e.m. (*n* = 6). **P < 0.01, significantly different between the two groups. (ANOVA with Tukey-Kramer post hoc test). (**c**) Cells were transfected with *Slc9a3r1*(−200/+168)::Luc and *Slc9a3r1* (+35/+168)::Luc in the absence or presence of *Per2* expression plasmids. Each value represents the mean with s.e.m. (*n* = 6). **P < 0.01, significant difference between the two groups (*F*_11,60_ = 58.935; *P* < 0.001; ANOVA with Tukey-Kramer post hoc test). (**d**) Enhancement of the transcriptional activity of *Slc9a3r1*(−200/+168)::Luc in *Per2* mutant (*Per2*^*m/m*^) cells. Mouse embryonic fibroblasts (MEFs) were prepared from wild-type and *Per2*^*m/m*^ mice. Cells were transfected with *Slc9a3r1*(*−*200/+168)::Luc or *Slc9a3r1* (+35/+168)::Luc. Each value represents the mean with s.e.m. (*n* = 6). **P < 0.01, significant difference between the two groups (*F*_3,20_ = 27.200; *P* < 0.001; ANOVA with Tukey-Kramer post hoc test). (**e**) The down-regulation of PER2 by miRNA enhances the transcriptional activity of *Slc9a3r1*(−200/+168)::Luc. The left panel shows a representative photograph of the immunoblot analysis of the PER2 protein in miRNA-expressing vector-transfected cells. Each value represents the mean with s.e.m. (*n* = 6). **P < 0.01, significant difference between the two groups (*F*_3,20_ = 516.818; *P* < 0.001; ANOVA with Tukey-Kramer post hoc test). (**f**) Cells were transfected with luciferase-reporter vectors containing the consensus NF-кB response element (NF-κB-RE::Luc), *Slc9a3r1* (−200/+168)::Luc, or *Slc9a3r1* (+35/+168)::Luc in the absence or presence of expression plasmids encoding p65, p50, or PER2. Each value represents the mean with s.e.m. (*n* = 6). **P < 0.01, significantly different from the control group; ^##^*P* < 0.01, significantly different from the p65/p50-transfected group (*F*_3,20_ = 127.370; *P* < 0.001 for *Slc9a3r1*(−200/+168)::Luc; *F*_3,20_ = 68.062; *P* < 0.001 for NF-κB-RE::Luc; ANOVA with Tukey-Kramer post hoc test). Full-size images of western blotting are presented in Supplementary Figs 10 and 11.

Although sequence analysis of 5000 base pairs (bp) up- and down-stream of the transcription start site of the mouse *Slc9a3r1* gene revealed no consensus DNA sequence of clock gene response elements, a highly conserved NF-κB-response element (NF-κB-RE) was located between +18 and +27 bp downstream from the transcription-start site (relative to the transcription start site,+1). This sequence was also identified at a similar position in the *Slc9a3r1* genes of rats, monkeys, and humans (Supplementary Fig. 1). Since several mechanisms have been suggested in the regulation of the NF-κB signaling pathway by circadian clock genes^19,20^, we focused on NF-κB-RE and performed transient transcription assays by constructing *Slc9a3r1* luciferase (Luc)-reporter vectors containing the NF-κB-RE of the mouse *Slc9a3r1* gene spanning from −200 to +168 bp. The reporter constructs *Slc9a3r1*(−200/+168)::Luc were co-transfected with expression vectors encoding CLOCK/BMAL1, PER1, PER2, PPARα/RXRα, RORα, REV-ERBα, DBP, or E4BP4. These expression vectors sufficiently increased the levels of clock gene products and modulated E-box-, PPRE-, RORE-, or D-site-driven transcriptional activity (Fig. 1a,b). Although the transcriptional activity of *Slc9a3r1*(−200/+168)::Luc was not significantly altered by CLOCK/BMAL1, PER1, PPARα/RXRα, RORα, REV-ERBα, DBP, or E4BP4, co-transfection of *Slc9a3r1* (−200/+168)::Luc with *Per2*-expressing vectors significantly decreased transcriptional activity (*P* < 0.01). The trans-repressive effects of PER2 on *Slc9a3r1* reporters disappeared following the deletion of the sequence up to bp +35 (Fig. 1c).

To further investigate the role of PER2 in the transcriptional regulation of *Slc9a3r1*, we prepared embryonic fibroblasts from wild-type and *Per2* mutant (*Per2*^*m/m*^) mice. Cells were transfected with *Slc9a3r1*(−200/+168)::Luc or *Slc9a3r1* (+35/+168)::Luc. Although the transcriptional activity of *Slc9a3r1*(+35/ +168)::Luc was not significantly different between wild-type and *Per2*^*m/m*^ cells, the activity of *Slc9a3r1* (−200/+168)::Luc in *Per2*^*m/m*^ cells was significantly higher than that in wild-type cells (*P* < 0.01, Fig. 1d). Furthermore, the down-regulation of PER2 in cultured hepatic cells by miRNA resulted in increases in the transcriptional activity of *Slc9a3r1*(−200/+168)::Luc (*P* < 0.01, Fig. 1e), but not of *Slc9a3r1*(+35/+168)::Luc. These results suggest that PER2 represses the expression of *Slc9a3r1* by acting on the DNA sequence between bp −200 and +35.

The classically-activated form of the NF-κB signal molecule consists of the v-rel reticuloendotheliosis viral oncogene homolog A (p65)/p50 heterodimer, which is typically the most abundant of the transactivating complexes^21^. Co-transfection of *Slc9a3r1* (−200/+168)::Luc with p65- and p50-expressing vectors significantly increased promoter activity (*P* < 0.01; Fig. 1f), although the transactivation effect was repressed by PER2 (*P* < 0.01). Furthermore, *Per2*-expressing vectors also suppressed the p65/p50-mediated transactivation of the Luc reporters containing consensus NF-κB-REs (Fig. 1f). These results suggest that PER2 acts as a repressor of p65/p50-mediated *Slc9a3r1* transactivation.

### Dysfunction in PER2 alters the diurnal rhythm of *Slc9a3r1*/NHERF1 in mouse liver

Next we investigated whether the expression of *Slc9a3r1* mRNA and its encoded protein NHERF1 were altered in the liver of *Per2*^*m/m*^ mice. To achieve this, male wild-type and *Per2*^*m/m*^ mice were maintained under a 12-h light-dark cycle (ZT, zeitgeber time; ZT0, lights on; ZT12, lights off) or constant dark conditions with food and water *ad libitum*. *Per2*^*m/m*^ mice harboring a deletion of 87 amino acids from the PER-ARNT-SIM (PAS) domain of the PER2 protein exhibited disrupted behavioral rhythms (Supplementary Fig. 2a). Mutated PER2 protein (lacking amino acid residues 348–434) exhibits difficulties translocating into the nucleus and, instead, accumulates in the cytoplasm^22,23^. The abundance of the mutated PER2 protein remained lower in the hepatic nuclear fraction of *Per2*^*m/m*^ mice than in that of wild-type mice throughout the day, without showing significant time-dependent oscillations under both light/dark cycle and constant dark conditions (Supplementary Fig. 2b).

**Figure 2:**
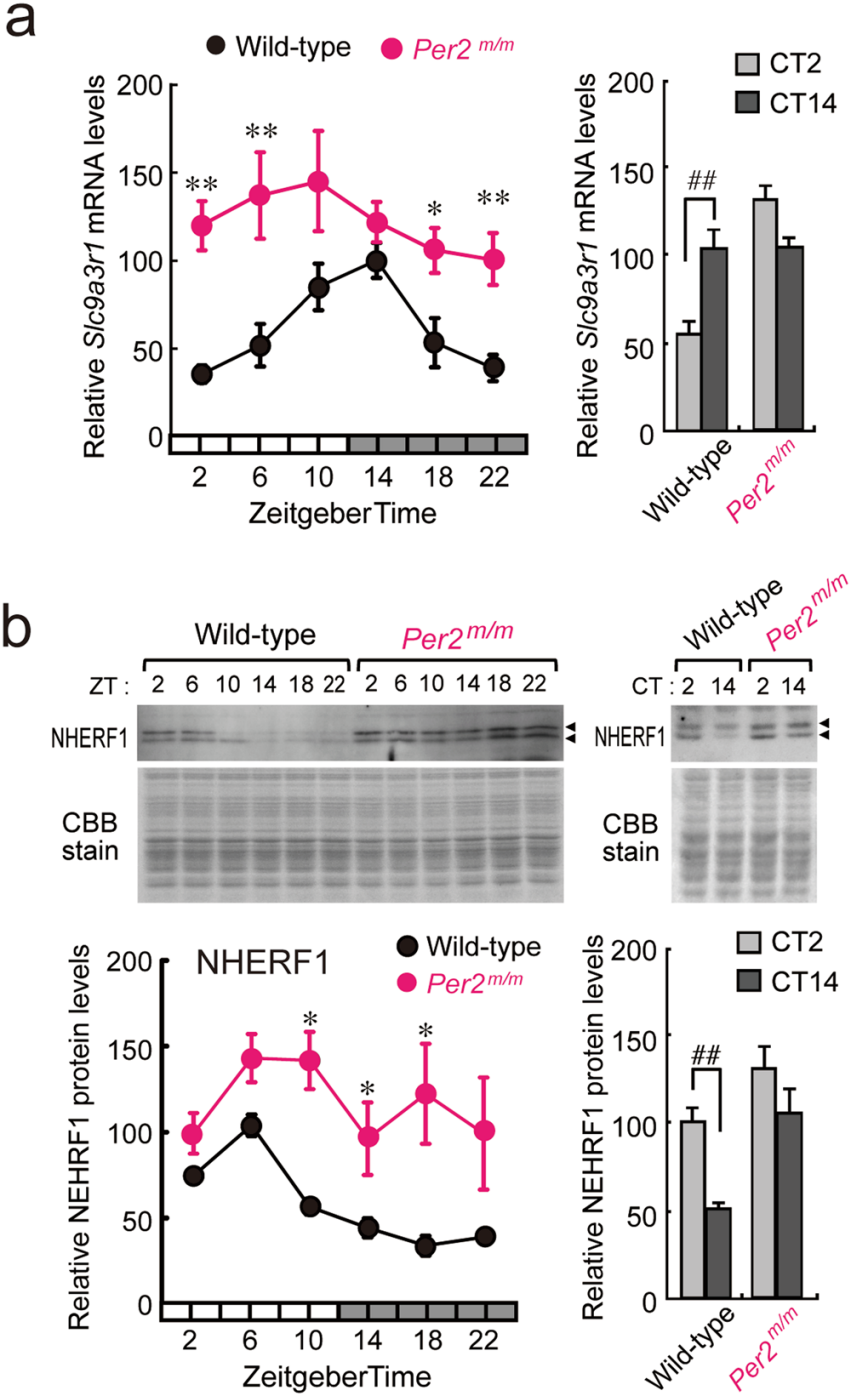
(**a**) Temporal-expression profiles of *Slc9a3r1* mRNA in the livers of wild-type and *Per2*^*m/m*^ mice maintained under a 12-h light/dark cycle (left) and constant dark condition (right). Each value represents the mean with s.e.m. of results for 4–6 mice. There were significant time-dependent variations in *Slc9a3r1* mRNA levels in wild-type mice (*F*_5,30_ = 5.829; *P* < 0.001; ANOVA). **P < 0.01; *P < 0.05, significantly different from wild-type mice at the corresponding time points (*F*_11,92_ = 5.764; *P* < 0.001; ANOVA with Tukey-Kramer post hoc test). ^##^*P* < 0.01, significant difference between the two groups (*F*_3,12_ = 37.113, *P* < 0.001; ANOVA with Tukey-Kramer post hoc test). (**b**) Temporal-expression profiles of NHERF1 protein in the hepatic plasma membrane of wild-type and *Per2*^*m/m*^ mice maintained under a 12-h light/dark cycle (left) and constant dark conditions (right). Each value represents the mean with s.e.m. for data from of 4–10 mice. There were significant time-dependent variations in the protein levels of NHERF1 (*F*_5,54_ = 28.402; *P* < 0.001; ANOVA). *P < 0.05, significantly different from wild-type mice at the corresponding time points (*F*_11,119_ = 5.618; *P* < 0.001; ANOVA with Tukey-Kramer post hoc test). ^##^*P* < 0.01, significant difference between the two groups (*F*_3,12_ = 37.113, *P* < 0.001; ANOVA with Tukey-Kramer post hoc test). For both panels, the mean peak values in wild-type mice were set at 100. Full-size images of immunoblotting are presented in Supplementary Fig. 12.

The expression of *Slc9a3r1* mRNA exhibited significant diurnal oscillations in the livers of wild-type mice maintained under light/dark cycle conditions (*P* < 0.01, Fig. 2a). *Slc9a3r1* mRNA levels peaked from the late light phase to the early dark phase. A similar time-dependent variation in *Slc9a3r1* mRNA expression was also detected in the livers of wild-type mice maintained under constant dark conditions (*P* < 0.01, Fig. 2a). Under light/dark cycle conditions, the plasmalemmal expression of NHERF1 in the livers of wild-type mice showed a significant diurnal oscillation (*P* < 0.01, Fig. 2b). Oscillations in NHERF1 protein levels in the hepatic membrane were delayed by approximately 12 h relative to the *Slc9a3r1* mRNA rhythm. In the livers of wild-type mice, the peak time for the accumulation of PER2 (Supplementary Fig. 2b) corresponded to the trough time of the *Slc9a3r1* mRNA rhythm (Fig. 2a). The reverse relationship between the accumulation of PER2 and mRNA expression of *Slc9a3r1* was also detected in the livers of wild-type mice maintained under constant dark conditions (Supplementary Fig. 2b and Fig. 2a). In contrast to wild-type mice, *Slc9a3r1* mRNA expression in the livers of *Per2*^*m/m*^ mice increased at all time points examined, with no significant diurnal variations (Fig. 2a). A similar disruptive effect of the *Per2* mutation was also detected in NHERF1 expression (Fig. 2b), with NHERF1 protein levels in the livers of *Per2*^*m/m*^ mice being elevated throughout the day. These results suggest that PER2 acts as a transcriptional repressor of the mouse *Slc9a3r1* gene, and that this repression is relieved in *Per2*^*m/m*^ mice, resulting in elevated levels of *Slc9a3r1* mRNA and its encoding protein NHERF1.

### Time-dependent repression of p65/p50-mediated transactivation of *Slc9a3r1* by PER2

**Figure 3:**
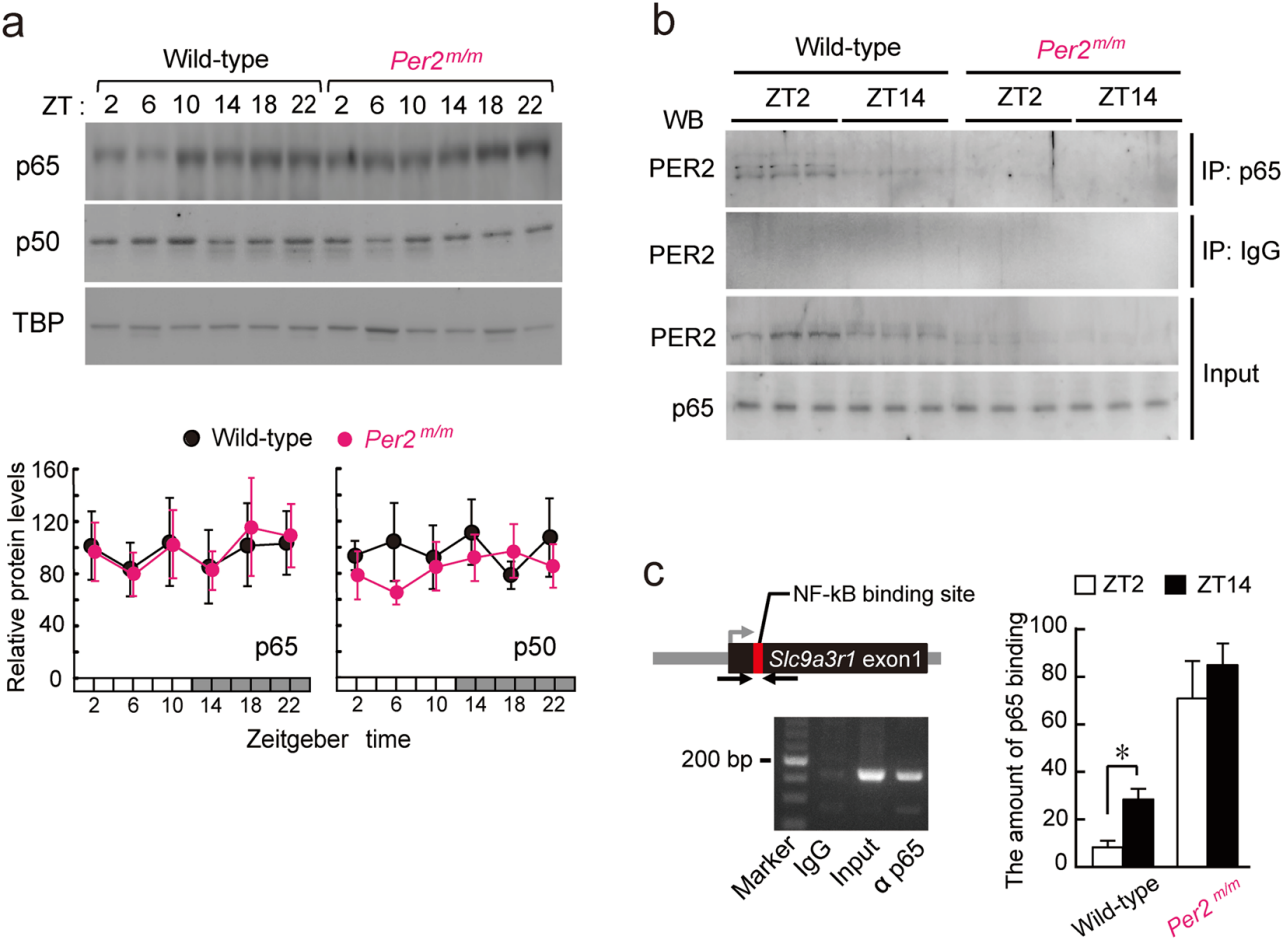
Interaction of PER2 with p65 underlies time-dependent changes in p65 binding to the *Slc9a3r1* promoter. (**a**) Temporal-expression profiles of p65 and p50 in hepatic nuclear fractions of wild-type and *Per2*^*m/m*^ mice maintained under a 12-h light/dark cycle. Each value represents the mean with s.e.m. for data from 6 mice. (**b**) Time-dependent interaction between PER2 and p65 in liver nuclei of wild-type and *Per2*^*m/m*^ mice. Nuclear extracts prepared at ZT2 and ZT14 were immunoprecipitated (IP) with antibodies against p65 and separated by SDS-PAGE. The blot was incubated with antibodies against PER2 or p65. (**c**) Temporal-binding profiles of p65 to the *Slc9a3r1* promoter in liver of wild-type and *Per2*^*m/m*^ mice maintained under a 12-h light/dark cycle. Cross-linked chromatin from livers collected at ZT2 and ZT14 were immunoprecipitated with antibodies against p65. Solid arrows represent PCR amplification areas containing the NF-kB-binding site. Each value represents the mean with s.e.m. for results from 3 mice. *P < 0.05, significant difference between the two time points (*F*_3,8_ = 4.500; *P* = 0.039; ANOVA with Tukey-Kramer post hoc test). Full-size images of immunoblotting are presented in Supplementary Figs 13 and 14.

The p65 and p50 protein levels in the hepatic nuclear fraction of wild-type and *Per2*^*m/m*^ mice did not show significant diurnal oscillations (Fig. 3a), and mutation of the *Per2* gene also had a negligible effect on p65 and p50 protein levels. Because the protein-protein interaction between p65 and another circadian clock protein has been demonstrated in the liver nuclei of mice^19,20^, we investigated whether PER2 bound to p65. The results of immunoprecipitation experiments using anti-p65 antibodies revealed that p65 immunoprecipitated together with PER2 in the nuclear fraction of the liver of wild-type mice, and that the amount of PER2 associated with p65 was much greater at ZT2 as compared with that observed at ZT14 (Fig. 3b). Similar time-dependent differences in the association of p65 with PER2 were also observed in immunoprecipitation experiments using anti-PER2 antibodies (Supplementary Fig. 3). Furthermore, chromatin-immunoprecipitation analysis of the liver of wild-type mice also demonstrated that p65 binding to the promoter region of the *Slc9a3r1* gene containing the NF-κB-RE at ZT14 was significantly enhanced compared to that observed at ZT2 (*P* < 0.05, Fig. 3c). In contrast to wild-type mice, the amount of p65 binding to the *Slc9a3r1* promoter in the liver of *Per2*^*m/m*^ mice was elevated in both light and dark phases (Fig. 3c). These results suggest that the time-dependent interaction between PER2 and p65 in the hepatic nuclear fraction underlies diurnal expression of *Slc9a3r1*/NHERF1.

### NHERF1 time-dependently interacts with FATP5 in hepatic cells

**Figure 4:**
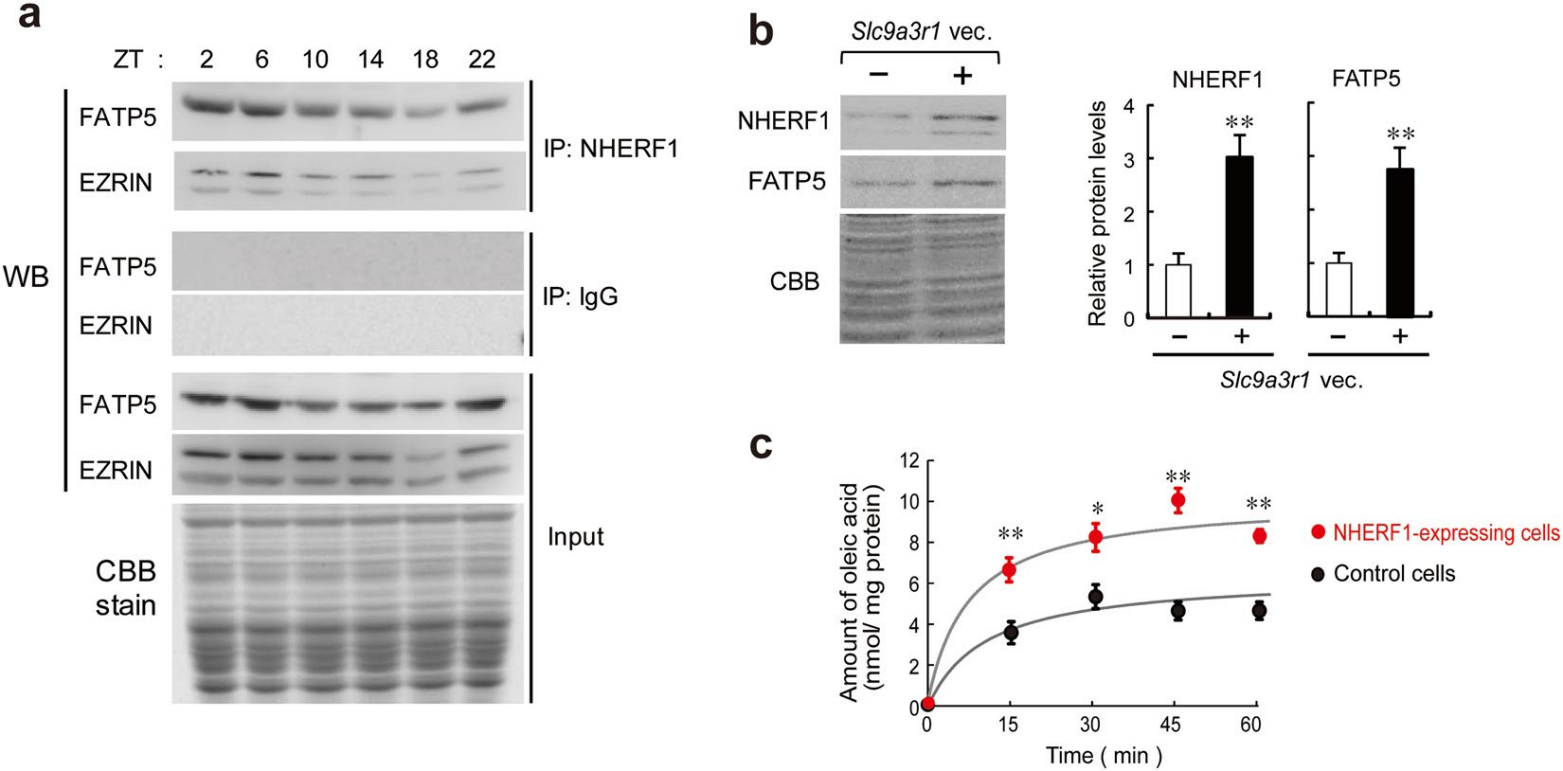
NHERF1 time-dependently interacts with FATP5 in the hepatic membrane fraction. (**a**) Western blot analysis of FATP5 and EZRIN in immunoprecipitates (IPs) from hepatic membrane fractions using anti-NHERF1 antibodies. (**b**) FATP5 protein levels on the plasma membrane prepared from NHERF1-expressing Hepa1–6 cells. Cells were transfected with plasmid vectors encoding *Slc9a3r1* (*Slc9a3r1* vec.). Control groups were transfected with empty vectors (pcDNA3.1) instead of expression plasmids. Presence (+) or absence (-) of expression plasmids (0.5 μg each) is denoted. Coomassie Brilliant Blue (CBB) indicates the equal loading of proteins from the membrane fraction. Each value represents the mean with s.e.m. (*n* = 6). **P < 0.01, significant difference from control group (*t*_10_ = 6.130 for FATP5; *t*_10_ = 3.919 for NHERF1; unpaired *t* test, two-sided). (**c**) The transporting activity of FATP5 in NHERF1-expressing Hepa1–6 cells. Each value represents the mean with s.e.m. (*n* = 6). **P < 0.01; *P < 0.05, significant difference from control cells at the corresponding times (*F*_5,50_ = 48.556; *P* < 0.001; ANOVA with Tukey-Kramer post hoc test). Full-size images of immunoblotting are presented in Supplementary Figs 15 and 16.

NHERF1 functions as a scaffold protein and plays important roles in the anchorage of various cell-surface proteins for their localization in plasma membranes^16,17^. Because the hepatic expression of NHERF1 in wild-type mice showed a significant diurnal oscillation (Fig. 2b), we searched for cell-surface proteins whose expression was time-dependently anchored by NHERF1. Hepatic membrane fractions were prepared from wild-type mice at ZT6 and ZT18. They were immunoprecipitated together with anti-NHERF1 antibodies, and the immunoprecipitates were analyzed by a negative gel stain. Despite using the same amount of anti-NHERF1 antibodies at both time points, the intensities of several protein bands showed time-dependent variations (Supplementary Fig. 4). Therefore, we further analyzed these proteins using liquid chromatography-mass spectrometry (LC-MS/MS). The analyses identified several proteins, including EZRIN, known as a NHERF1-interacting protein, and the fatty acid transporter FATP5 which was a novel candidate NHERF1-interacting protein (Supplementary Fig. 4). The time-dependent interaction of NHERF1 with FATP5 was confirmed by western blot analysis of immunoprecipitates from hepatic membrane fractions using anti-NHERF1 antibodies (Fig. 4a). A similar result was also obtained in immunoprecipitation experiments using anti-EZRIN and anti-FATP5 antibodies (Supplementary Fig. 5).

In order to obtain direct evidence to show that NHERF1 stabilizes the plasmalemmal localization of FATP5, we assessed FATP5 protein levels in the plasma membranes of hepatic cells stably expressing NHERF1. Although the total protein levels of FATP5 were not significantly altered in NHERF1-expressing cells (Supplementary Fig. 6), the plasmalemmal localization of FATP5 was significantly increased and accompanied by elevations in NHERF1 protein levels (*P* < 0.01; Fig. 4b). As expected, we also observed enhancements in the uptake of oleic acid, a typical FATP5 substrate^24^, in hepatic cells stably expressing NHERF1 (Fig. 4c). These results indicate that NHERF1 stabilizes the plasmalemmal localization of FATP5 and also enhances its function of transporting fatty acids.

### Disruption of the time-dependency of plasmalemmal localization of FATP5 in *Per2*^*m/m*^ mice

**Figure 5:**
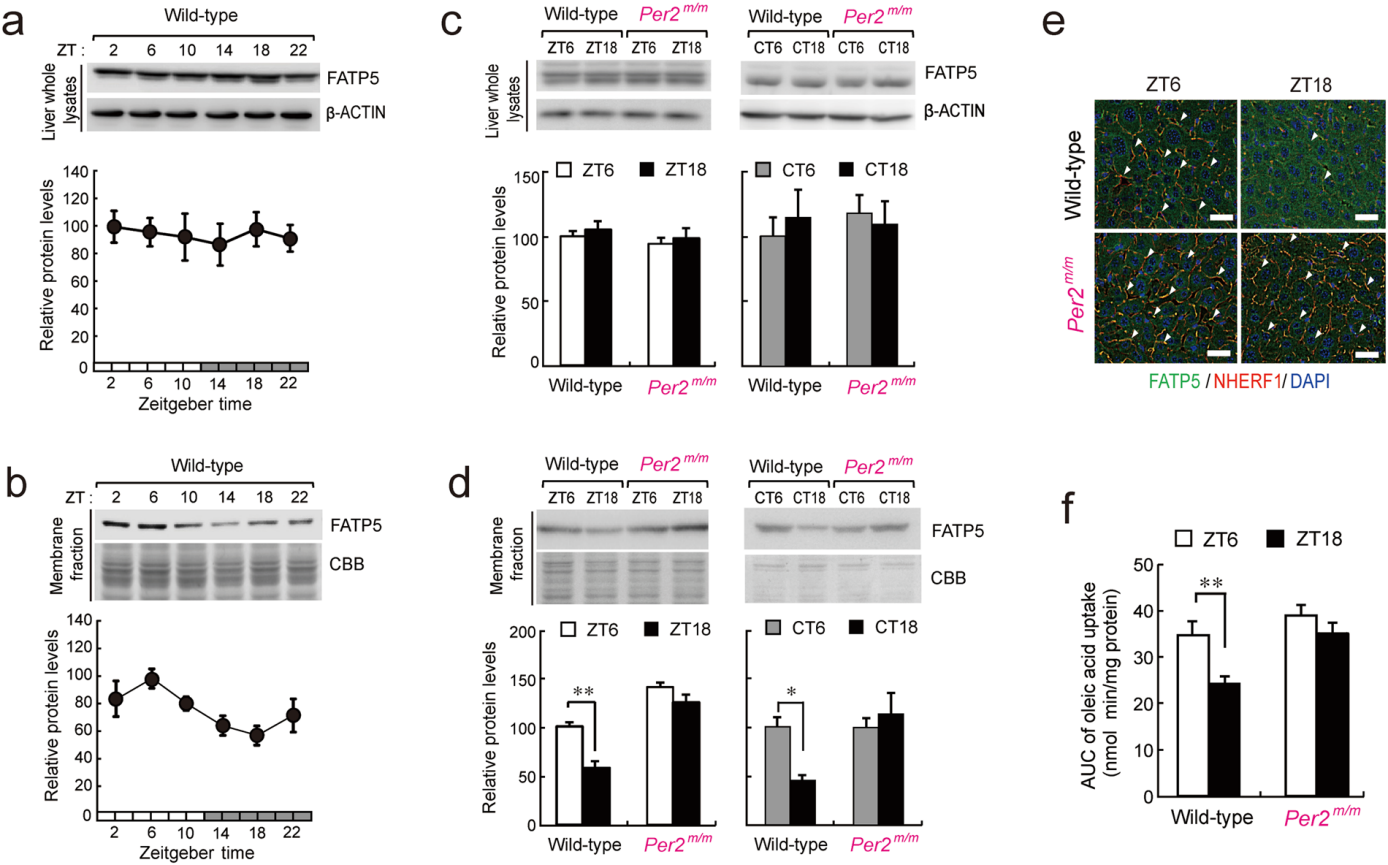
PER2 controls the time-dependent expression of FATP5 in the hepatic membrane fraction. (**a**,**b**) Temporal expression profiles of the FATP5 protein in whole-liver lysates (**a**) and membrane fractions (**b**) prepared from wild-type mice maintained under a 12-h light/dark cycle condition. The mean peak values of FATP5 protein levels were set to 100. Each value represents the mean with s.e.m. for data from 6–10 mice. There were significant 24-h variations in FATP5 protein levels in the hepatic membrane fraction. (*F*_5,54_ = 28.402; *P* < 0.001; ANOVA). (**c**,**d**) Temporal expression profiles of FATP5 protein in whole-liver lysates (**c**) and membrane fractions (**d**) prepared from wild-type and *Per2*^*m/m*^ mice maintained under a 12-h light/dark cycle (left) and constant dark (right) conditions. The mean peak values of FATP5 protein levels from wild-type mice were set to 100. Each value represents the mean with s.e.m. for data from 6 mice. **P < 0.01, *P < 0.05, significant difference between the two groups (*F*_3,12_ = 5.546; *P* < 0.05; ANOVA with Tukey-Kramer post hoc test, right panel). (**e**) Co-immunofluorescent staining of FATP5 (green) and NHERF1 (red) in the livers of wild-type and *Per2*^*m/m*^ mice maintained under a 12-h light/dark cycle. Yellow signals indicate the co-localization of FATP5 and NHERF1. Liver sections were prepared at ZT6 and ZT18. Nuclei were also stained with 4′6-diamidino-2-phenylindole (DAPI; blue). Scale bars indicate 10 µm. Data were collected for more than three mice in each group. (**f**) Temporal profile of the transporting activity of FATP5. Data show time-dependent differences in the area under the curve of the amount of oleic acid uptake into liver slices prepared from wild-type and *Per2*^*m/m*^ mice maintained under a 12-h light/dark cycle. The uptake of oleic acid was assessed for 30 min. Each value represents the mean with s.e.m. for data from 6 mice. **P < 0.01, significant difference between the two groups (*F*_3,20_ = 12.222; *P* < 0.001; ANOVA with Tukey-Kramer post hoc test). In panels a, b, c, and d, full-size images of immunoblotting are presented in Supplementary Figs 17 and 18.

Since the diurnal expression of NHERF1 was disrupted in the liver of *Per2*^*m/m*^ mice, we also investigated whether disruption of circadian clock function affects the plasmalemmal localization of FATP5. Under both light/dark cycle conditions, there was no significant time-dependent variation in FATP5 protein levels in whole-liver lysates prepared from wild-type mice (Fig. 5a), whereas FATP5 expression in the hepatic membrane fraction showed significant diurnal oscillation, with a peak from the early to middle light phase (*P* < 0.01, Fig. 5b). Similar time-dependent variation in the expression of FATP5 was also detected in the hepatic membrane fraction of wild-type mice maintained under constant dark conditions (*P* < 0.01, Fig. 5d). The rhythmic phase of plasmalemmal localization of FATP5 in the liver of wild-type mice corresponded to NHERF1 protein rhythm (Fig. 2b). Although the protein levels of FATP5 in the whole liver lysates of *Per2*^*m/m*^ mice were similar to those observed in wild-type mice (Fig. 5c), its plasmalemmal localization in *Per2*^*m/m*^ mice increased under both light/dark cycle and constant dark conditions (Fig. 5d). Consistent with these results, immunofluorescent histochemical analyses of wild-type liver sections also revealed that NHERF1 and FATP5 were co-localized on the plasma membrane during the light phase (ZT6, Fig. 5e, upper panels), but this co-localization was not obviously detected during the dark phase (ZT18). On the other hand, NHERF1 and FATP5 were co-located on the hepatic plasma membrane of *Per2*^*m/m*^ mice at both light and dark phases (Fig. 5e, lower panels).

The amount of oleic acid taken up into hepatic slices prepared from wild-type mice at ZT6 was significantly higher than that observed in those prepared at ZT18 (*P* < 0.01, Fig. 5f and Supplementary Fig. 7). The ability of hepatic slices to uptake oleic acid was enhanced at the time of day during which plasmalemmal localization of FATP5 was abundant. In contrast, no significant time-dependent variations were detected in the uptake of oleic acid by hepatic slices prepared from *Per2*^*m/m*^ mice (Fig. 5f). The ability of hepatic *Per2*^*m/m*^ slices to uptake oleic acid was elevated during both light and dark phases. Taken together, these results suggest that dysfunction of PER2 disrupts diurnal localization of FATP5 on the plasma membrane, probably through the arrhythmic expression of NHERF1.

## Discussion

**Figure 6:**
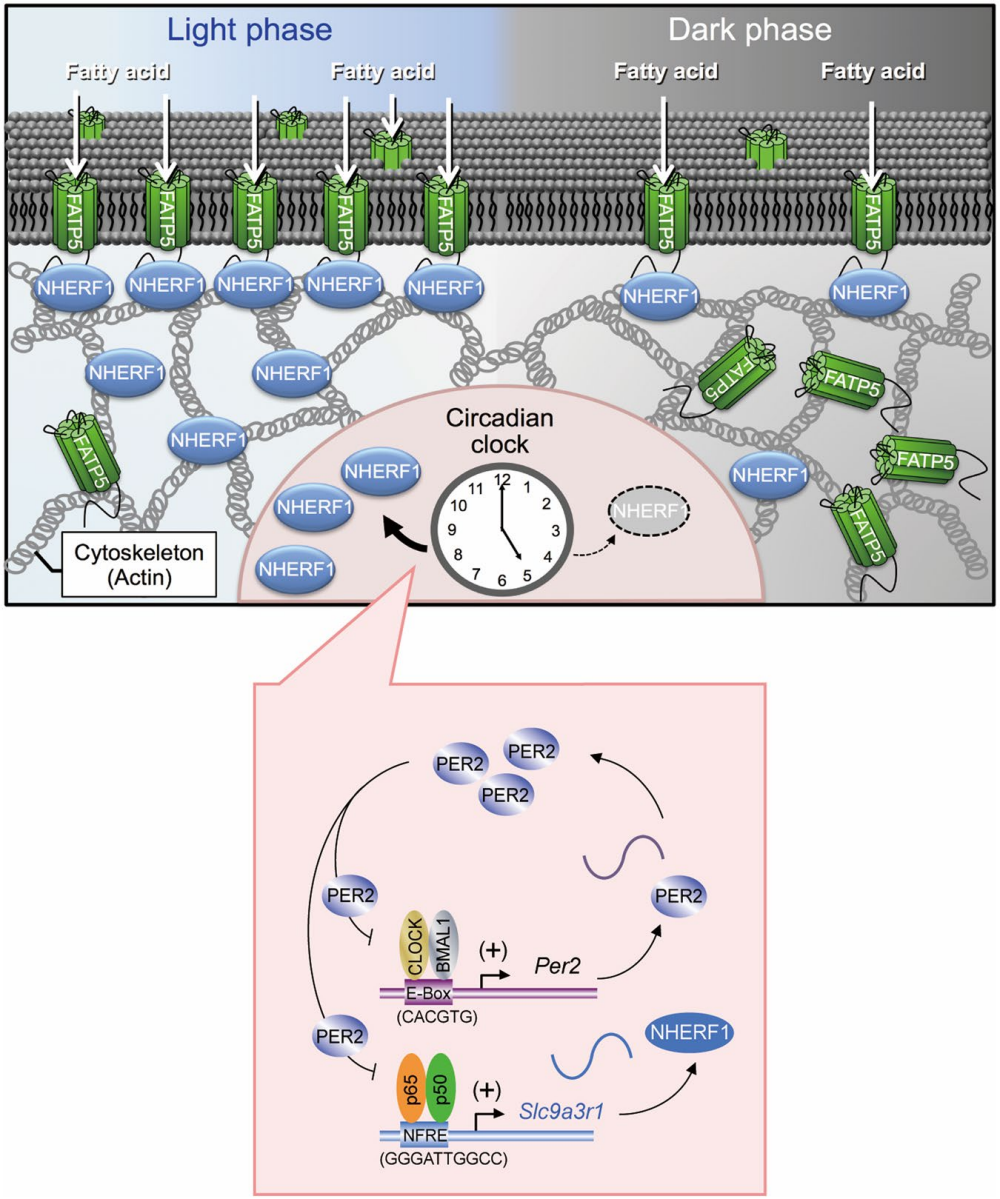
Schematic diagrams indicating NHERF1-regulated diurnal expression of FATP5 on hepatic plasma membrane. The expression of *Slc9a3r1*/NHERF1 is under the control of a circadian clock. The p65/p50-mediated transcription of *Slc9a3r1* is periodically repressed by PER2, thereby generating diurnal oscillation in the expression of NHERF1 protein. Temporal elevations in NHERF1 protein levels induce the plasmalemmal localization of FATP5 in hepatic cells during certain times of the day, which enhances the uptake of fatty acids into hepatic cells.

The circadian clock machinery controls various downstream events through transcription, translation, or degradation processes. The results of the present study demonstrated that the core circadian clock component PER2 caused the time-dependent expression of *Slc9a3r1*/NHERF1 in the livers of mice through periodic repression of p65/p50-mediated transactivation. Temporary accumulation NHERF1 protein in the hepatic cells enhanced plasmalemmal localization of FATP5, thereby causing diurnal oscillation in its transporting function (Fig. 6).

The time-dependent interaction between PER2 and p65 appeared to prevent the p65/p50-mediated transactivation of *Slc9a3r1*. Under inflammatory conditions, there are two distinct pathways for the activation of NF-κB signaling. The canonical pathway activates complexes associated with p65 and p50, thereby inducing the expression of genes responsible for inflammation and cell survival^25^. In contrast, an alternative pathway involving RelB/p52 complexes controls lymphogenesis and B-cell maturation^26,27^. Although inappropriate regulation of NF-κB activity has been implicated in the pathogenesis of several inflammatory diseases, accumulating evidences have also suggested that NF-κB regulates the expression of genes outside of the immune system, and also maintains multiple aspects of normal physiology^28,29^. In the present study, we also detected considerable expression of p65 and p50 in the nuclear fractions of healthy mouse liver. The abundance of p65 and p50 proteins in the hepatic nuclear fractions of both wild-type and *Per2*^*m/m*^ mice was constant throughout the day, suggesting that basal expression of *Slc9a3r1* is dependent on transcriptional activation by p65/p50.

A previous ChIP sequencing analysis revealed that PER2 binds to a site bp +1801 downstream of the putative transcriptional start site of the *Slc9a3r1* gene^30^, although no clock gene response elements were located around the region. We also performed a transient transcription assay by constructing *Slc9a3r1* luciferase-reporter vectors that contained the downstream region of the mouse *Slc9a3r1* gene spanning from bp +1600 to +2000. Reporter constructs *Slc9a3r1*(+1600/+2000)::Luc were co-transfected with expression vectors encoding PER2. The transfection of cells with *Per2*-expressing vectors increased PER2 protein levels; however, the transcriptional activity of *Slc9a3r1*(+1600/+2000)::Luc was not affected by elevation of PER2 (Supplementary Fig. 8). Although other *Slc9a3r1* genomic regions may also be responsive to PER2 protein, the trans-repressive effects of PER2 on NF-κB-RE near the transcriptional start site appeared to be associated with the diurnal expression of *Slc9a3r1* mRNA and its encoded protein, NHERF1.

In the circadian core feedback loop, PER2 acts as a repressor of CLOCK/BMAL1- mediated transactivation; however, this circadian repressor protein also inhibits gene expression by interacting with non-circadian clock proteins^31–33^. *Per2*^*m/m*^ mice produce a mutated PER2 protein, which lacks two exons encoding PAS domains^22^ and exhibits reduced nuclear localization, accumulating, instead, in the cytoplasm^23^. The PAS domain is highly conserved in the products of circadian clock genes in a number of species, and is implicated in the mediation of protein-protein interactions^34^. Due to the inability of the mutated PER2 protein to interact with other clock proteins, *Per2*^*m/m*^ mice exhibit abnormal circadian properties in terms of physiology and behavior^22^. An immunoprecipitation experiment using hepatic nuclear fractions prepared from wild-type mice revealed that PER2 interacted with p65. The interaction of these proteins was enhanced at the time of day when the amount of p65 binding to the promoter region of *Slc9a3r1* was decreased. Although we still have not clarified whether PER2 directly binds to p65, PER2 appeared to prevent *Slc9a3r1* transcription by forming complexes with p65. Therefore, it is likely that PER2 represses *Slc9a3r1* promoter activity without decreasing p65 and p50 protein levels in liver nuclei.

Small amounts of the mutated PER2 proteins were detected in the hepatic nuclear fraction prepared from *Per2*^*m/m*^ mice, but the protein levels failed to show significant diurnal oscillations. In addition, we were also unable to detect the formation of complexes between mutated PER2 proteins and p65 in liver nuclei of *Per2*^*m/m*^ mice. A conservative interpretation of these results is that negating the repressive function of PER2 on p65 in liver nuclei allows for constant binding of p65 to the promoter region of *Slc9a3r1*, thereby increasing NHERF1 expression throughout the day. On the other hand, a previous study has reported that the CLOCK protein also binds to p65 and enhances NF-κB-signaling activity via p65 acetylation^19^. PER2 forms complexes with the CLOCK protein through the PAS domain interaction^35^ and serves as a co-repressor by ordering the recruitment of repressive chromatin modifiers^36,37^. The interaction between PER2 and CLOCK interferes with the intrinsic transacetylase activity of CLOCK^38^, and, consequently, PER2 might also repress *Slc9a3r1* transcription by preventing CLOCK-mediated p65 acetylation.

A study using yeast two-hybrid screening suggests that NHERF1 binds to various xenobiotic transporters, including organic cation/carnitine transporter (OCTN)1, OCTN2, organic anion transporter (OAT) 4, and OAT polypeptide A^39^. NHERF1 also facilitates the sorting and stabilization of multidrug resistance-associated protein-2 in rat liver hepatocytes^17,40^. In the present study, we identified a novel NHERF1-interacting protein, FATP5, which is exclusively expressed in the liver of rodents and humans^24^. In whole hepatic cells, FATP5 was constantly expressed throughout the day, whereas its amount varied in the hepatic plasma membrane according to the time of day. Since NHERF1 stabilized the expression of FATP5 in the plasma membrane, the diurnal accumulation of NHERF1 protein appeared to cause the time-dependent localization of FATP5 in the hepatic plasma membrane, thereby enhancing the hepatic uptake of fatty acids at certain times of the day.

The physiological role of this hepatic transporter involves the uptake of long-chain fatty acids into hepatocytes^24,41^. Mice deficient in FATP5 show not only decreases in the hepatic uptake of fatty acids, but also their disrupted distribution among other organs^24^. In contrast, elevations in the hepatic expression of FATP5 are implicated in non-alcoholic fatty liver disease^42^. A previous study indicates that PER2 is involved in the regulation of fatty acid metabolism and the pro-adipogenic activity of peroxisome proliferator-activated receptor-γ^43^. Consequently, mice harboring a mutated *Per2* gene exhibit enhanced oxidative capacity in white adipose tissues due to the upregulation of genes responsible for lipid metabolism^44^. We observed the disruption of diurnal changes in the hepatic uptake of fatty acids in *Per2*^*m/m*^ mice. The constant elevations of fatty acid uptake in hepatic cells in *Per2*^*m/m*^ mice may also contribute to their enhanced lipid metabolism.

In general, protein levels are regulated at nearly any stage of the gene expression process. In addition to their synthesis, mRNAs and proteins are both also subject to degradation, which influences the abundance of membrane proteins. The circadian expression of clock and clock-controlled proteins occurs at the transcriptional, post-transcriptional, translational, or post-translational levels^44,45,46^. The results of the present study suggest a previously unacknowledged role for circadian machinery regulating the plasmalemmal expression of a cell-surface transporter protein. The time-dependent accumulation of NHERF1 appeared to stabilize the plasmalemmal localization of FATP5 by constituting the fundamental building blocks of membrane-protein complexes. Because NHERF1 has been suggested to interact with a number of diverse cell-surface proteins, this scaffold protein may also be involved in the diurnal regulation of plasmalemmal localization of other plasma membrane proteins and their functions in hepatic cells.

## Experimental Procedures

### Animals and treatments

*Per2*^*m/m*^ mice with an ICR background and wild-type mice of the same strain were housed six per cage in a temperature-controlled room (24 ± 1 °C) under a 12-h light/dark cycle (ZT0, light on; ZT12 light off) with food and water *ad libitum*. In mice housed under constant dark conditions, the circadian time (CT) was used instead of ZT; and CT0 was characterized by the beginning of the subjective light phase, while CT12 was defined as the beginning of the subjective dark phase. During the dark period, a dim red light was used to aid animal treatment. Mouse embryonic fibroblasts were prepared by standard techniques^47^ from the litter-mate embryos of *Per2*^*m/m*^ or wild-type mice, and cells were maintained in Dulbecco’s modified Eagle’s medium (DMEM, Sigma-Aldrich, St. Louis, MO) supplemented with 10% fetal bovine serum (FBS) and 0.5% penicillin-streptomycin at 37 °C in a humidified 5% CO_2_ atmosphere. All animal experiments followed the Law for the Humane Treatment and Management of Animals and other related laws and regulations. All the experiments were conducted under a protocol approved by the internal committee for animal experiment in Kyushu University.

### Cell culture

NIH3T3 and Hepa1-6 cells were purchased from the Cell Resource Center for Biomedical Research, Tohoku University (Sendai, Japan) and RIKEN BioResource Center (Tsukuba, Japan), respectively. Cells were maintained in DMEM supplemented with 10% FBS and 0.5% penicillin-streptomycin at 37 °C in a humidified 5% CO_2_ atmosphere.

### Construction of reporter and expression vectors

To investigate the ability of expression vectors encoding circadian clock genes, we constructed luciferase reporter vectors containing four tandem repeats of E-box (CACGTG), PPRE (AGGTCAAAGGTCA), RORE (AAAGTAGGTCA), or D-site (TTATGTAA). The annealed oligonucleotides that contain tandem repeats of each response element were inserted into pGL4.12 reporter vectors (Promega). The mouse *Slc9a3r1*-promoter region spanning from −200 to +168 bp was amplified by polymerase chain reaction (PCR), and the products were ligated into the pGL4.12 luciferase-reporter vector (Promega, Madison, WI, USA) using DNA ligation reagents (Takara, Otsu, Japan). Deletion constructs of *Slc9a3r1* (−200)::Luc were prepared by removing the regions starting at 35 bases [*Slc9a3r1*(+35/+168)::Luc] downstream of the transcription-start site of the mouse *Slc9a3r1* gene. Expression vectors for mouse CLOCK, BMAL1, PER1, PER2, PPARα, RXRα, RORα, REV-ERBα, DBP, E4BP4, p65, and p50 were constructed using cDNA generated from mouse liver RNA by reverse-transcription (RT)-PCR. All coding regions were ligated into the pcDNA3.1(+) vector (Invitrogen; Life Technologies, Carlsbad, CA, USA).

### Luciferase reporter assay

NIH3T3 cells were seeded at a density of 1 × 10^4^/well in 24-well culture plates. Cells were transfected with 100 ng of the pGL4.12 reporter construct and 2 µg (total) of expression vectors. The pcDNA3.1 empty vector was added to adjust the total amount of DNA in all transfections. A total of 10 ng of the pRL-TK vector (Promega) was also transfected as an internal-control reporter. Cells were harvested 24 h after transfection, and lysates were analyzed using the Dual-Luciferase reporter assay system (Promega). The ratio of firefly (expressed from the pGL4.12 reporter construct) to Renilla (expressed from pRL-TK) luciferase activities in each sample served as a measure of normalized luciferase activity.

### Western blot analysis

The preparation of membrane fractions from mouse liver was conducted by ultracentrifugation, as described previously^48^. Livers were homogenized in Krebs-Ringer buffer [11.7 mM NaCl, 4.7 mM KCl, 1.2 mM MgCl_2_, 1.2 mM NaH_2_PO_4_·2H_2_O, 25 mM NaHCO_3_, 2.5 mM CaCl_2_·2H_2_O, and 11 mM d-(+)-glucose] containing appropriate protease inhibitors (2 µg/mL leupeptin, 2 µg/mL aprotinin, and 100 µM phenyl- methanesulfonyl fluoride). Mouse liver lysates were then separated by centrifugation at 8,000 × *g* at 4 °C for 15 min, and the supernatant obtained was ultracentrifuged at 100,000 × *g* at 4 °C for 1 h. Pellets were resuspended in MOPS-Tris buffer [20 mM 4-morpholine propanesulfonic acid–Tris (pH 7.0), 300 mM sucrose, 5 mM EDTA, and protease inhibitors] and ultracentrifuged at 100,000 × *g* at 4 °C for 1 h. The pellets obtained were then resuspended in Tris-EDTA buffer [10 mM Tris-HCl, 5 mM EDTA (pH 7.0), and protease inhibitors]. Preparation of plasma membranes was confirmed by blotting for the cell-surface marker cadherin (Supplementary Fig. 9). Hepatic nuclear fractions were prepared using a LysoPure nuclear and cytoplasmic extractor kit (Wako Chemicals, Osaka, Japan). Denatured samples containing 20 or 40 µg of each protein fraction were separated by sodium dodecyl sulfate polyacrylamide gel electrophoresis (SDS-PAGE) and transferred to a polyvinylidene difluoride membrane. Separated proteins were stained with Coomassie Brilliant Blue as a control for equal loading of the membrane fraction. Membranes were reacted with antibodies against CLOCK (1:1000; D349–3, Medical & Biological Laboratories; MBL, Nagoya, Japan), BMAL1 (1:1000; sc-48790, Santa Cruz Biotechnology), PER1 (1:1000; sc-7724, Santa Cruz Biotechnology), PER2 (1:1000; sc-25363, Santa Cruz Biotechnology), PPARα (1:1000; sc-9000, Santa Cruz Biotechnology), RXRα (1:1000; sc-553, Santa Cruz Biotechnology), RORα (1:1000; sc28612, Santa Cruz Biotechnology), REV-ERBα (1:1000; sc-135241, Santa Cruz Biotechnology), DBP (1:4000; PM079, MBL), E4BP4 (1:1000; sc-9549, Santa Cruz Biotechnology), NHERF1 (1:1000; ab3452; Abcam, Cambridge, UK), FATP5 (1:1000; sc-70172; Santa Cruz Biotechnology), pan-Cadherin (ab16505, 1:4000; Abcam), β-ACTIN (1:1000; sc-1616; Santa Cruz Biotechnology), p65 (1:1000; ab16502; Abcam), p50 (1:1000; ab32360; Abcam), and TATA-binding protein TBP (1:1000; ab51841; Abcam). Specific antigen-antibody complexes were visualized using horseradish peroxidase-conjugated anti-rabbit (1:10,000; sc-2004; Santa Cruz Biotechnology), anti-goat IgG (1:10,000; sc-2020; Santa Cruz Biotechnology), and anti-mouse IgG (1:10,000; sc-2005; Santa Cruz Biotechnology) antibodies and ImmunoStar LD (Wako Chemicals). Visualized images were scanned using an ImageQuant LAS4000 (GE Healthcare, Ltd., Little Chalfont, UK).

### Construction of miRNA expression vectors

In order to down-regulate the expression of the PER2 protein in Hepa1–6 cells, we constructed miRNA expression vectors using a BLOCK-iT Pol II miRNA-Expression Vector Kit (Invitrogen; Life Technologies). The miRNA oligonucleotide against the *Per2* gene (anti-*Per2* miRNA) or control oligonucleotide against *LacZ* (control) was annealed at 95 °C for 4 min and then ligated to pcDNA 6.2-GW/miR vector. The target sequences of *Per2* and *LacZ* are listed in Supplementary Table 1.

### Real-time PCR analysis

Total RNA was extracted from mouse livers using RNAiso Plus (Takara) according to the manufacturer’s instructions. cDNAs were synthesized by reverse transcription using a ReverTra Ace qPCR RT Kit (Toyobo, Osaka, Japan). The cDNA equivalent of 10 ng of RNA was amplified by PCR using a 7500 real-time PCR system (Applied Biosystems; Life Technologies) with THUNDERBIRD SYBR qPCR Mix (Toyobo). The sequences of the forward and reverse primers were as follows: mouse *Slc9a3r1* (NCBI reference sequence: NM_012030.2) forward: 5′-CCTCCAGCGATACCAGTGAG-3′, *Slc9a3r1* reverse: 5′-CACAGCCAAGGAGATGTTGAG-3′; mouse *Slc27a5* (NCBI reference sequence: NM_009512.2) forward: 5′-GGCGTAACAGTGATCTTGTATGTG-3′, *Slc27a5* reverse: 5′-TCTTGTCTTCTGGTTGCTCAGG-3′; mouse β-Actin (NCBI reference sequence: NM_007393.5) forward: 5′-GGCTGTATTCCCCTCCATCG-3′, β-actin reverse: 5′-CCAGTTGGTAACAATGCCATGT-3′. Relative RNA levels were expressed as a percentage of the maximum value obtained for each experiment.

### Immunoprecipitation analysis

One milligram of proteins in hepatic nuclear or liver membrane fractions were reacted using a Crosslink immunoprecipitation kit (Thermo Fisher Scientific, Waltham, MA, USA). Three-hundred microliters of the lysate were pre-cleared with control protein A/G agarose and then incubated at 4 °C overnight with protein A/G agarose-binding anti-NHERF1 antibodies (1:500) or anti-p65 antibodies (1:500). After washing the reactants multiple times, immunoprecipitation lysates were denatured at 60 °C for 30 min with 0.1% TritonX-100, 1% SDS, 15% glycerol, 0.25 M Tris, and 5% 2-mercaptoethanol. Immunoprecipitates prepared from nuclear fractions were separated by SDS-PAGE and transferred to a polyvinylidene difluoride membrane. Immunoprecipitated proteins were detected by western blotting. Denatured samples of immunoprecipitates prepared from membrane fractions were separated by SDS-PAGE and stained with a negative gel stain using a Gel-Negative stain kit for SDS-PAGE (Nacalai Tesque, Kyoto, Japan) according to manufacturer’s instructions. Visualized images were scanned using an Image Quant LAS4000 (GE Healthcare).

### Chromatin immunoprecipitation (ChIP) analysis

Cross-linked chromatin from liver samples was sonicated on ice, and nuclear fractions were obtained by centrifugation at 10,000 × *g* for 5 min. Supernatants were incubated with antibodies against p65 (1:100; ab16502; Abcam), p50 (1:100; ab32360; Abcam), or rabbit IgG (1:100; sc66931; Santa Cruz Biotechnology). DNA was purified using a DNA purification kit (Promega) and amplified by PCR for the surrounding NF-κB-RE in the 5′-flanking region of the mouse *Slc9a3r1* gene. Primer sequences for amplification of the surrounding NF-κB-RE were as follows: mouse NF-κB-RE forward: 5′-GTCTACTGTGATCCACACC-3′; and NF-κB-RE reverse: 5′-AGAGCTCGAGTTGGGAG-3′. The quantitative reliability of PCR was evaluated by kinetic analysis of the amplified products to ensure that signals were derived only from the exponential phase of amplification. Chromatin immunoprecipitation proceeded in the absence of antibody or in the presence of rabbit IgG as negative controls.

### LC-MS/MS analysis

To identify proteins binding to NHERF1, the respective protein bands were cut from the gel and decolorized. Gel fragments were analyzed by nano-LC-MS (Japan Proteomics Co., Ltd., Sendai, Japan).

### Immunofluorescent histochemical staining

To investigate the plasmalemmal localization of NHERF1 and FATP5, immunofluorescent histochemical staining was performed using frozen liver sections as described previously^49^. Cryostat hepatic sections were fixed with 4% paraformaldehyde and incubated with primary antibodies at 4 °C for 12 h, followed by incubation with secondary antibodies (Cy3; Sigma-Aldrich) at 25 °C for 2 h. The stained slices were placed on glass slides and, after air-drying, the slices were mounted using Vectashield hard-set mounting medium with 4′,6-diamidino-2-phenylindole (Vector Laboratories, Burlingame, CA, USA). Visualized images were scanned using a BZ-9000 instrument (Keyence, Osaka, Japan).

### Construction of cells stably expressing NHERF1

In order to establish NHERF1-expressing cells, full-length mouse *Slc9a3r1* cDNA was subcloned into the BamHI and EcoRI sites of a pcDNA3.1 (+) vector (Invitrogen; Life Technologies), and the constructs were transfected into Hepa1–6 cells. Transgene-expressing cells were selected with G418 (Wako Chemicals), and individual colonies were then expanded and maintained in media containing 400 µg/mL G418. The up-regulation of NHERF1, encoded by the *Slc9a3r1* gene, was confirmed by western blotting, and NHERF1 activity was evaluated using [1-^14^C]-oleic acid (0.2 µCi/mL; specific activity: 1.48 × 10^8^ Bq/mmol).

### Assessment of oleic acid transport into hepatic cells

NHERF1 stably expressing Hepa1–6 cells were incubated in transport buffer containing 3.4 µM [1-^14^C]-oleic acid (2 µCi/mL; specific activity: 1.48 × 10^9^ Bq/mmol; Perkin Elmer, Wellesley, MA, USA) at 37 °C. Cells were washed twice with ice-cold phosphate-buffered saline and homogenized in 500 µL of ice-cold 6 M NaOH solution. After centrifugation, 300 µL of the supernatant was added to Clear-sol1 (Nacalai Tesque) for scintillation counting, and the remainder of the supernatant was used to measure protein concentrations (BCA protein assay; Takara).

### Statistical analysis

The values presented are expressed as means with standard error of the mean. The significance of the 24-h variations for each parameter was tested by analysis of variance (ANOVA). The statistical significance of differences among groups was analyzed by ANOVA, followed by Tukey-Kramer post hoc test. Equal variances were not formally tested. Values of *P* < 0.05 were considered significant. No statistical method was used to predetermine sample sizes; however, our sample sizes were similar to those reported in previous studies^8,23,28^. Experiments were not randomized.

### Data availability

All data supporting the results of the present study are included in the article, either in the main figures or as supplementary information files.

## Electronic supplementary material

Supplementary Information
