# Supplementary material for: Circadian clock component PERIOD2 regulates diurnal expression of Na+/H+ exchanger regulatory factor-1 and its scaffolding function
Source: Sci Rep. 2018 Jun 13;8:9072. doi: 10.1038/s41598-018-27280-w (PMC5998136; doi:10.1038/s41598-018-27280-w)
Supplement: Supplementary file 1 — Supplementary Information [file 41598_2018_27280_MOESM1_ESM.docx]

**Supplementary information**

**Circadian clock component PERIOD2 regulates diurnal expression of Na^+^/H^+^ exchanger regulatory factor-1 and its scaffolding function**

Yuya Tsurudome^1^, Satoru Koyanagi^1,2^, Takumi Kanemitsu^1^, Chiharu Katamune^1^, Masayuki Oda^1^, Yuki Kanado^1^, Mizuki Kato^1^, Akari Morita^1^, Yu Tahara^3,4^, Naoya Matsunaga^1,2^, Shigenobu Shibata^4^, Shigehiro Ohdo

1. Department of Pharmaceutics, Faculty of Pharmaceutical Sciences, Kyushu University, Fukuoka, Japan
2. Department of Glocal Healthcare Science, Faculty of Pharmaceutical Sciences, Kyushu University, Fukuoka, Japan
3. Department of Psychiatry and Behavioral Sciences, University of California, Los Angeles, 760 Westwood Plaza, CA 90024 U.S.A
4. Laboratory of Physiology and Pharmacology, School of Advanced Science and Engineering, Waseda University, Japan

Correspondence should be addressed to S.O. (ohdo@phar.kyushu-u.ac.jp)

**Contents**

Supplementary Figure 1 Alignment of DNA sequences of *Slc9a3r1* genes in mouse (mus musculus), rat (ratta norvegicus), monkey (macaca mulatta), and human (homo sapiens).

Supplementary Figure 2 Alterations in diurnal rhythms in locomotor activity and expression of PER2 protein in hepatic nuclei of *Per2*^m/m^ mice.

Supplementary Figure 3 Time-dependent interaction between PER2 and p65 in the liver nuclei of wild-type mice.

Supplementary Figure 4 Immunoprecipitation analysis of NHERF1-interacting proteins in the hepatic membrane fraction of mice.

Supplementary Figure 5 Time-dependent interaction of NHER1 with FATP5 and EZRIN in the liver nuclei of wild-type mice.

Supplementary Figure 6 FATP5 protein levels in whole-cell lysates prepared from NHERF1-expressing Hepa1-6 cells.

Supplementary Figure 7 Temporal profile of the transporting activity of FATP5.

Supplementary Figure 8 Effects of PER2 on the transcriptional activity of *Slc9a3r1* luciferase reporters containing the intron region of the mouse *Slc9a3r1* gene.

Supplementary Figure 9 Preparation of plasma membrane from the mouse liver.

Supplementary Figure 10 Unedited full blots of Supplementary Figure 1

Supplementary Figure 11 Unedited full blots of Figure 1e

Supplementary Figure 12 Unedited full blots of Figure 2b

Supplementary Figure 13 Unedited full blots of Figure 3a

Supplementary Figure 14 Unedited full blots of Figure 3b

Supplementary Figure 15 Unedited full blots of Figure 4a

Supplementary Figure 16 Unedited full blots of Figure 4b

Supplementary Figure 17 Unedited full blots of Figure 5a and 5b

Supplementary Figure 18 Unedited full blots of Figure 5c and d

Supplementary Figure 19 Unedited full blots of Supplementary Figure 2b

Supplementary Figure 20 Unedited full blots of Supplementary Figure 3

Supplementary Figure 21 Unedited full blots of Supplementary Figure 5

Supplementary Figure 22 Unedited full blots of Supplementary Figure 6

Supplementary Figure 23 Unedited full blots of Supplementary Figure 8

Supplementary Figure 24 Unedited full blots of Supplementary Figure 9

Supplementary Table 1 miRNA sequences for target gene

**
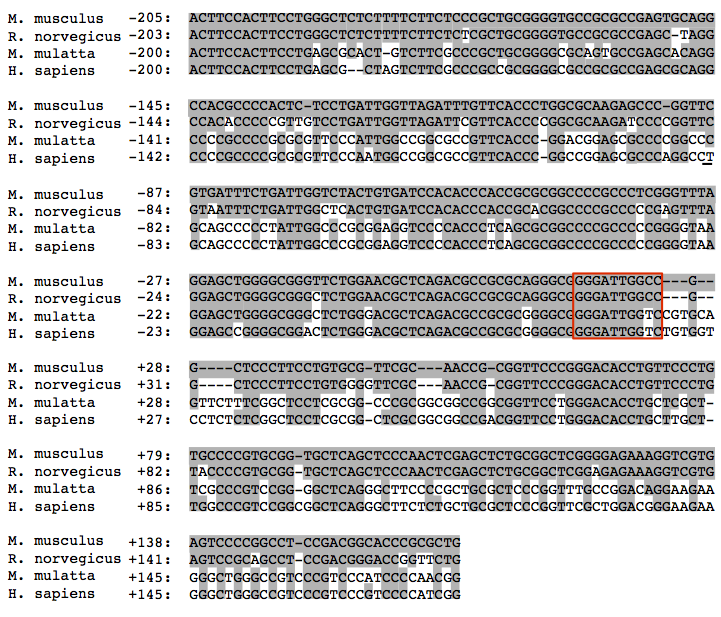
**

**Supplementary Figure 1** **Alignment of DNA sequences of *Slc9a3r1* genes in mouse (mus musculus), rat (ratta norvegicus), monkey (macaca mulatta), and human (homo sapiens).** Gray indicates consensus compared with the sequences of mus musculus. Consensus sequence of NF-κB response element indicates red square.

**
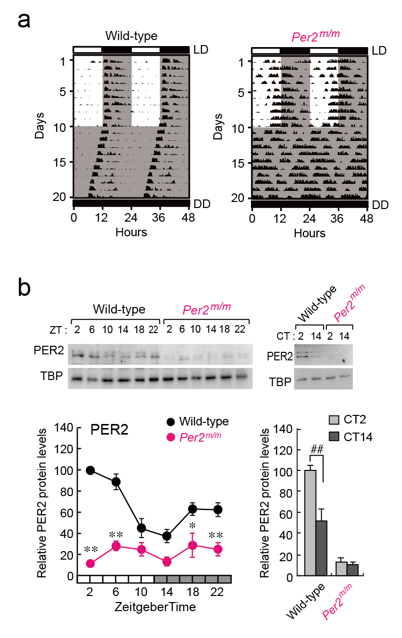
Supplementary Figure 2 Alterations in diurnal rhythms in locomotor activity and expression of PER2 protein in hepatic nuclei of *Per2*^m/m^ mice.** (a) Representative locomotor activity records of wild-type and *Per2^m/m^* mice. Light regime: 10 days in a 12-h light and 12-h dark cycle (LD) followed by 10 days in a constant dark cycle (DD). Mice were housed individually in breeding cages with food and water ad libitum. Cages were placed into an area with an infrared sensor, and locomotor activity was measured every 10 min. (b) Temporal-expression profiles of PER2 protein in the hepatic nuclei of wild-type and *Per2^m/m^* mice maintained under a 12-h light/dark cycle (left) and constant dark condition (right). Each value represents the mean with s.e.m. of 4–10 mice. There were significant time-dependent variations in the protein levels of PER2 (*F*_5,30_=6.598; *P*<0.001; ANOVA). ***P*<0.01; **P*<0.05, significantly different from wild-type mice at the corresponding time points (*F*_11,71_=19.765; *P*<0.001; ANOVA and Tukey-Kramer post hoc test). ^##^*P*<0.01, significant difference between the two groups (*F*_3,12_=37.113, *P*<0.001; ANOVA and the Tukey-Kramer post hoc test). Full-size images of western blotting are presented in Supplementary Fig.19.


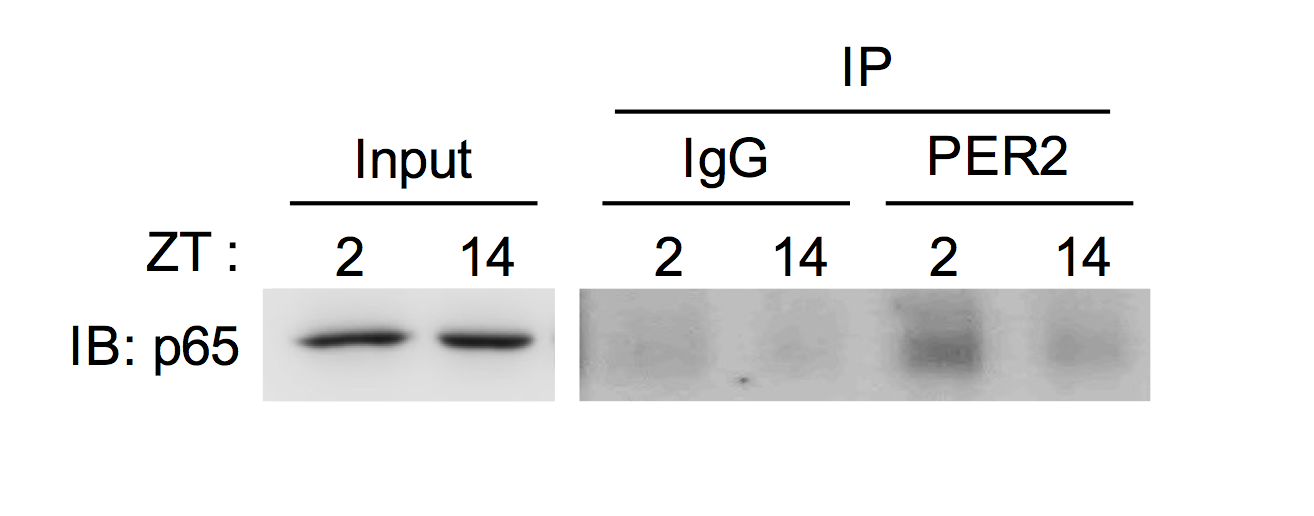
**Supplementary Figure 3 Time-dependent interaction between PER2 and p65 in the liver nuclei of wild-type mice**. Nuclear extracts prepared at ZT2 and ZT14 were immunoprecipitated (IP) with antibodies against PER2 and separated by SDS-PAGE. The blot was incubated with antibodies against p65. . Full-size images of western blotting are presented in Supplementary Fig.20.


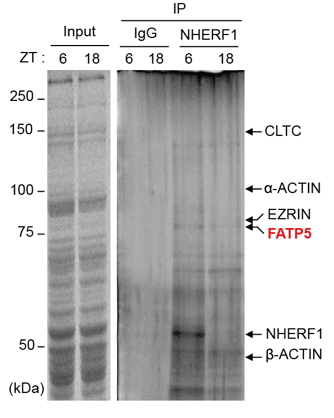


**Supplementary Figure 4 Immunoprecipitation analysis of NHERF1-interacting proteins in the hepatic membrane fraction of mice**. Plasma-membrane fractions were prepared from the liver of wild-type mice at ZT6 and ZT18 and then subjected to immunoprecipitation with an anti- NHERF1 antibody, followed by a negative gel stain. Coomassie brilliant blue indicates the equal loading of proteins. LC-MS/MS analysis was applied to examine the sequences of tryptic proteins that were time-dependently immunoprecipitated with NHERF1. All proteins listed on the right side represented significant results over the scoring threshold 37 (*P*<0.05). The allows were placed corresponding to the molecular weight of proteins that were detected by LC-MS/MS.


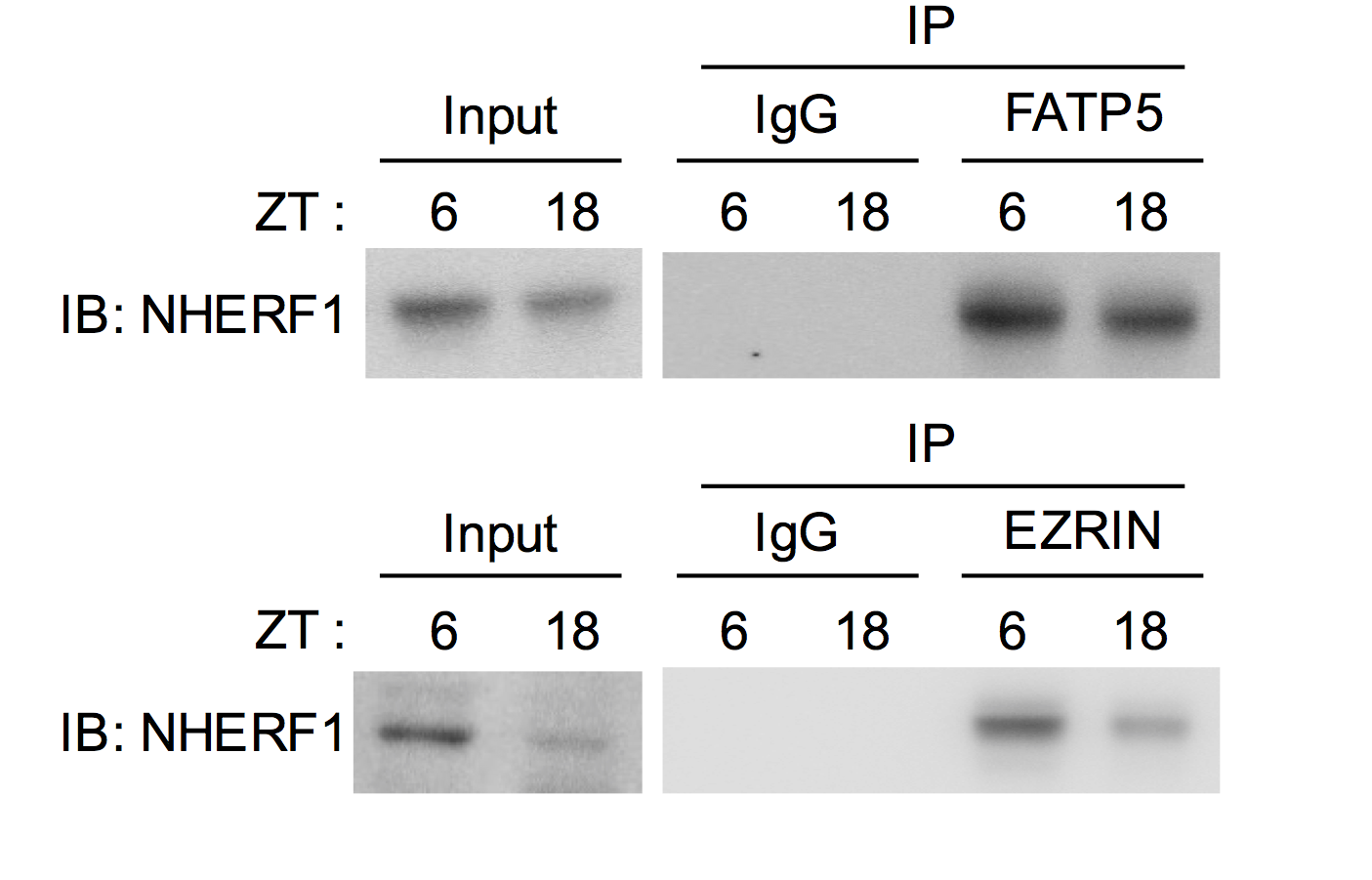


**Supplementary Figure 5 Time-dependent interaction of NHER1 with FATP5 and EZRIN in the liver nuclei of wild-type mice**. Nuclear extracts prepared at ZT6 and ZT18 were immunoprecipitated (IP) with antibodies against FATP5 (upper panel) or EZRIN (lower panel) and then separated by SDS-PAGE. The blot was incubated with antibodies against NHERF1. Full-size images of western blotting are presented in Supplementary Fig.21.


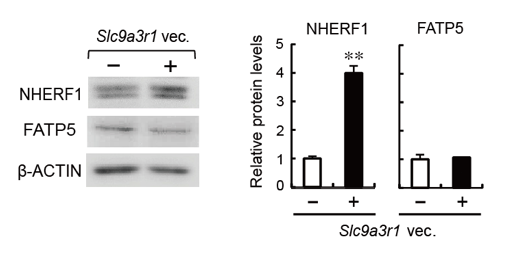


**Supplementary Figure 6 FATP5 protein levels in whole-cell lysates prepared from NHERF1-expressing Hepa1-6 cells.** Cells were transfected with plasmid vectors encoding *Slc9a3r1* gene (*Slc9a3r1* vec.). Each value represents the mean with s.e.m. (*n* = 6). ***P*<0.01, significantly different from the empty vector (pcDNA3.1)-transfected group (*t*_10_=3.953; unpaired *t* test, two-sided). Full-size images of western blotting are presented in Supplementary Fig.22.


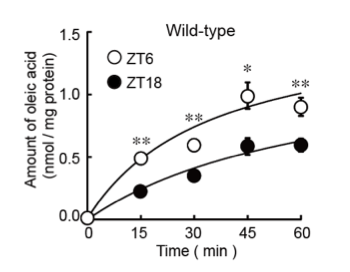


**Supplementary Figure 7** **Temporal profile of the transporting activity of FATP5.** The amount of oleic acid uptake was assessed using liver slices prepared from wild-type and *Per2^m/m^* mice at ZT6 and ZT18. The left panel shows the time course of oleic acid uptake into liver slices prepared from wild-type mice. Each value represents the mean ±s.e.m. (*n* = 6). ***P*<0.01; **P*<0.05, significantly different from the corresponding time points (*F*_9,50_ = 36.717, *P* < 0.001; ANOVA and Tukey-Kramer post hoc test).


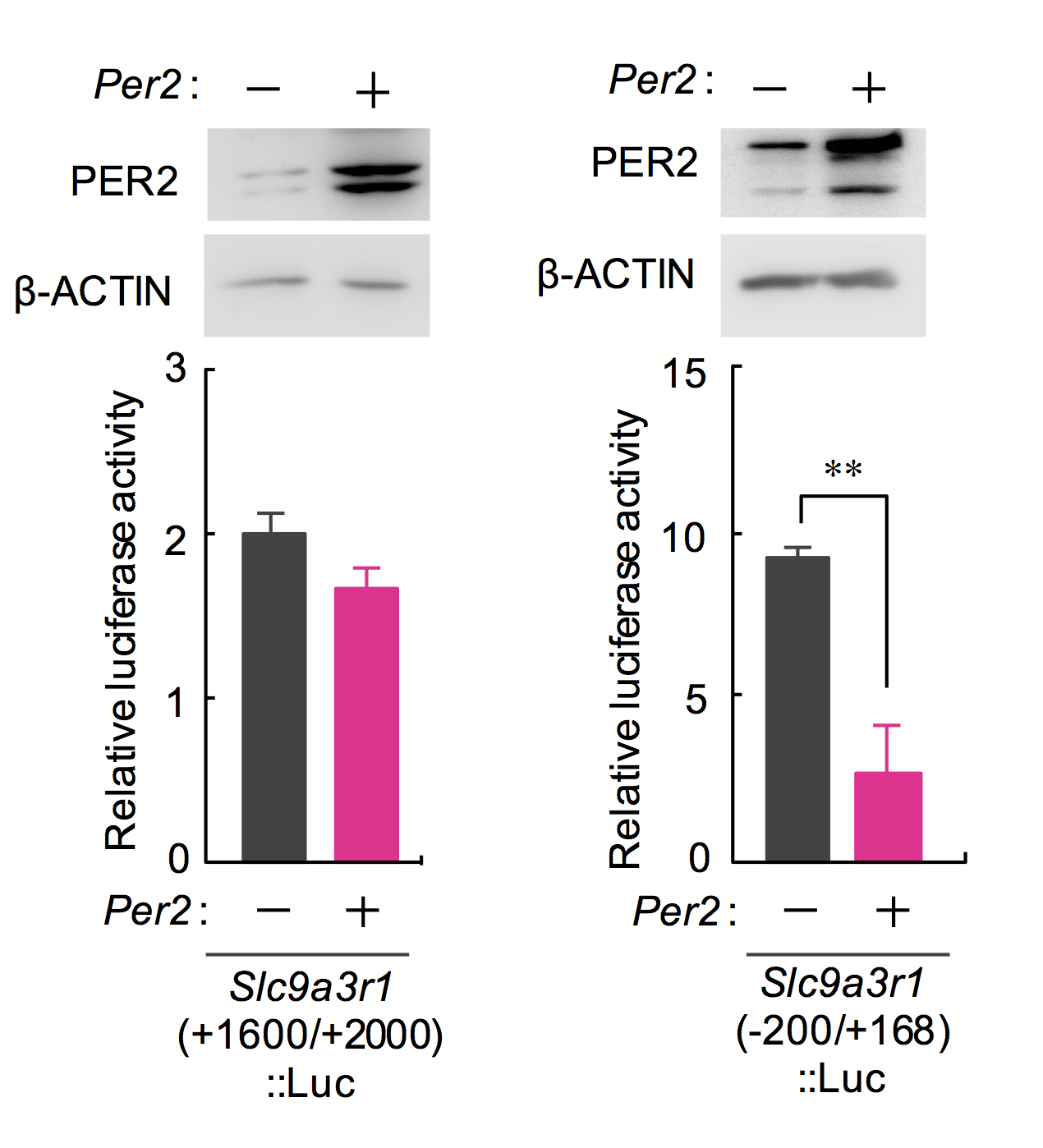
**Supplementary Figure 8** **Effects of PER2 on the transcriptional activity of *Slc9a3r1* luciferase reporters containing the intron region of the mouse *Slc9a3r1* gene.** Luciferase-reporter constructs containing the intron region of the mouse *Slc9a3r1* gene spanning from bp +1600 to +2000 (*Slc9a3r1* (+1600/+2000)::Luc) or *Slc9a3r1* (-200/+168)::Luc were transfected with expression plasmids encoding *Per2*. Control groups were transfected with empty vectors (pcDNA3.1) instead of expression plasmids. Presence (+) or absence (−) of expression plasmids (0.5 µg each) is denoted. Each value represents the mean ± s.e.m. (*n* = 3-6). ***P*<0.01, significantly different between the two groups. (*t*_10_=4.648; P<0.01 t-test). Luciferase activity was assessed 24 h after transfection. Full-size images of western blotting are presented in Supplementary Fig.23.


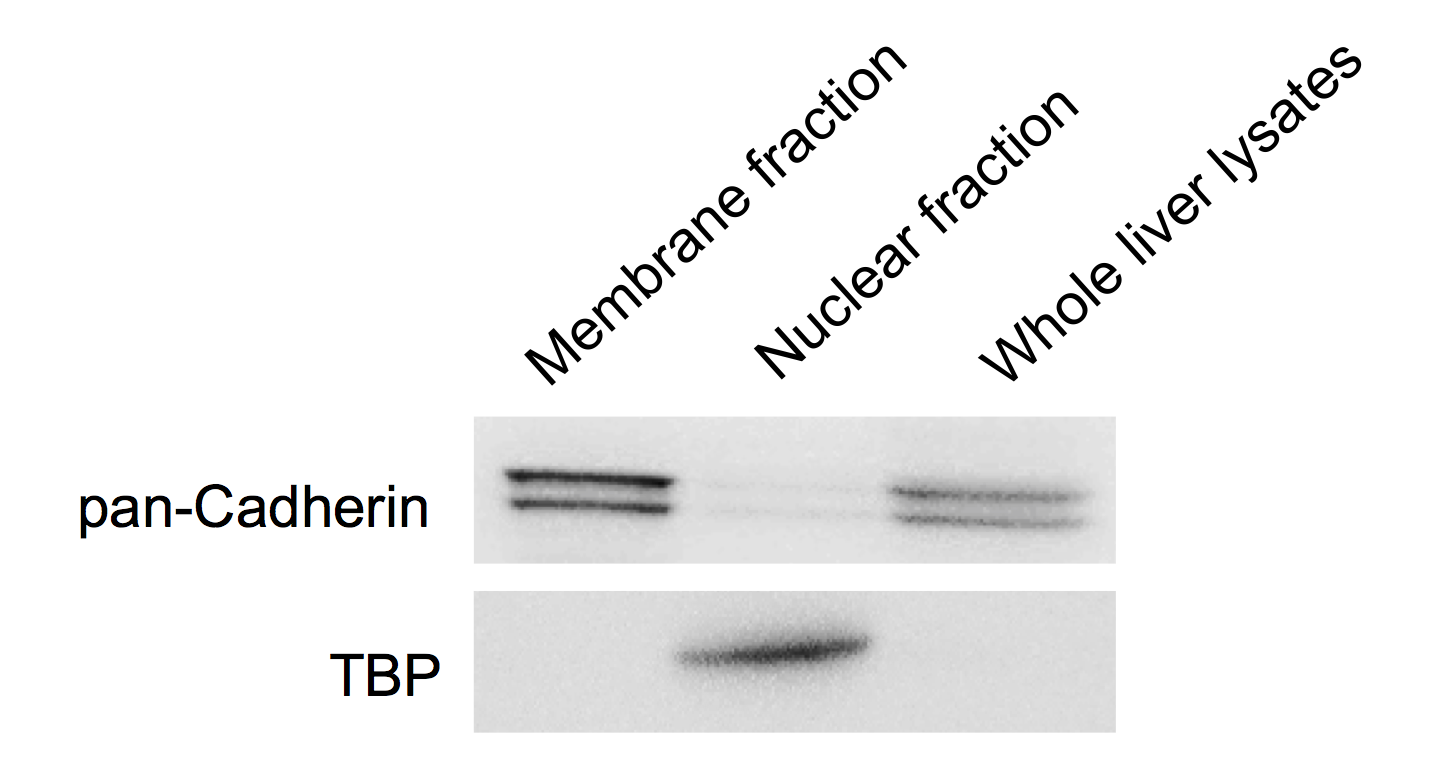


**Supplementary Figure 9 Preparation of plasma membrane from the mouse liver.** Membrane and nuclear fractions were prepared from the liver of wild-type mice. Denatured samples containing 20μg of each fraction protein were separated by SDS-PAGE, transferred to polyvinylidene difluoride membranes, and evaluated chemiluminescent Western blot for pan-Cadherin and TBP. Full-size images of western blotting are presented in Supplementary Fig.24.

**
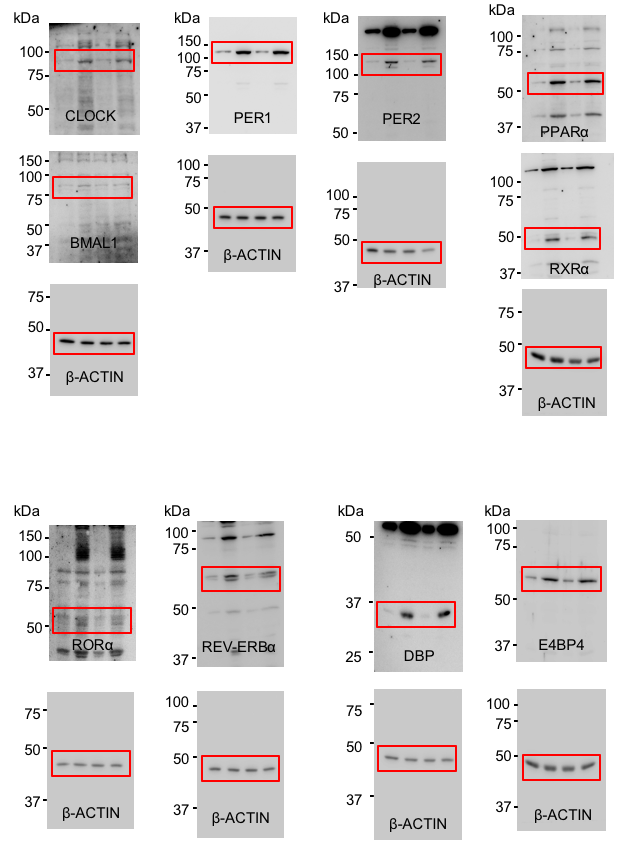
**

**Supplementary Figure 10** Unedited full blots of Supplementary Figure 1

**
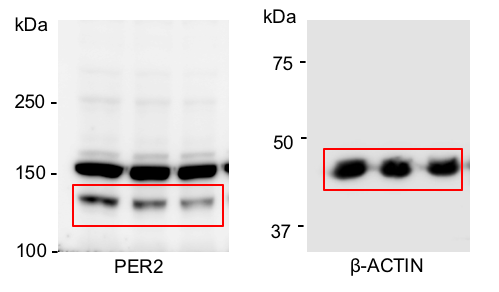
**

**Supplementary Figure 11** Unedited full blots of Figure 1e


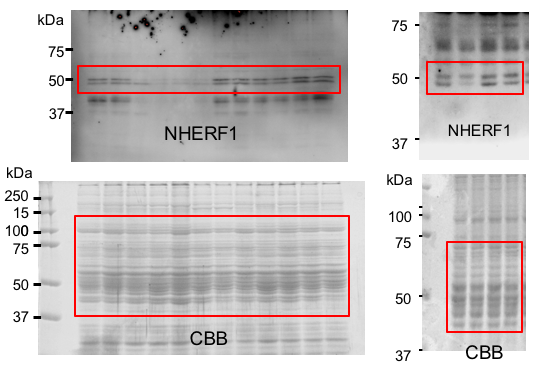


**Supplementary Figure 12** Unedited full blots of Figure 2b


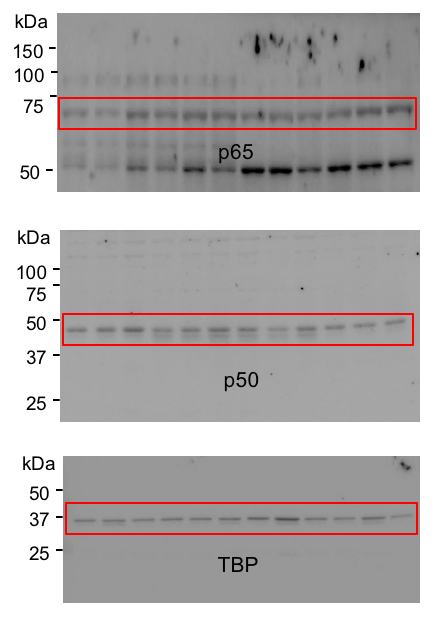


**Supplementary Figure 13** Unedited full blots of Figure 3a


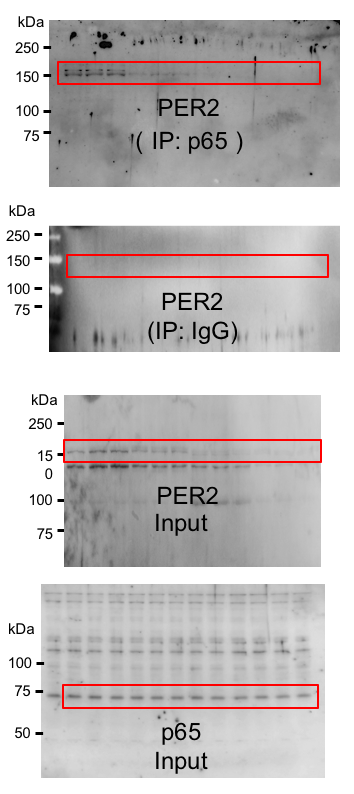


**Supplementary Figure 14** Unedited full blots of Figure 3b


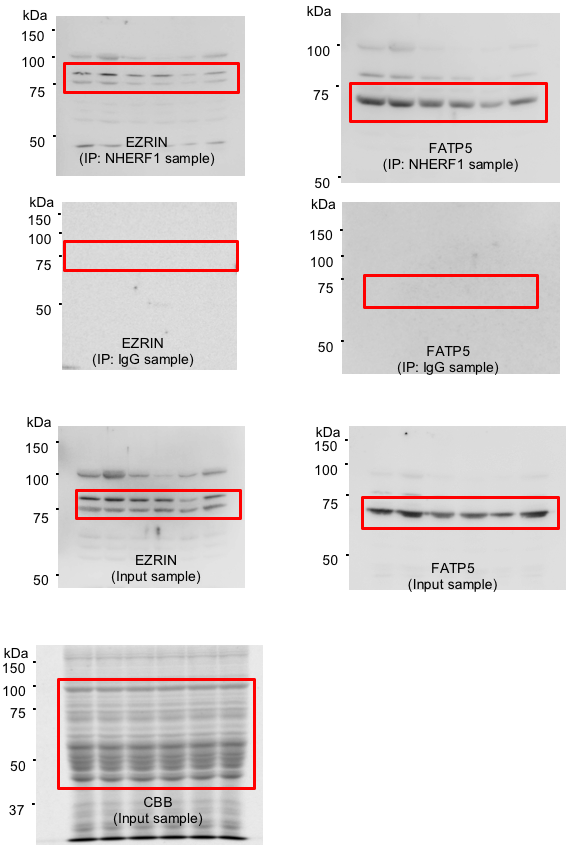


**Supplementary Figure 15** Unedited full blots of Figure 4a


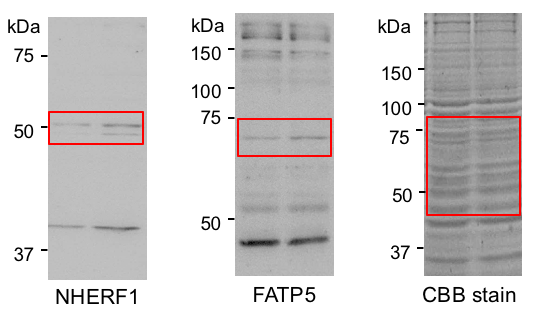


**Supplementary Figure 16** Unedited full blots of Figure 4b


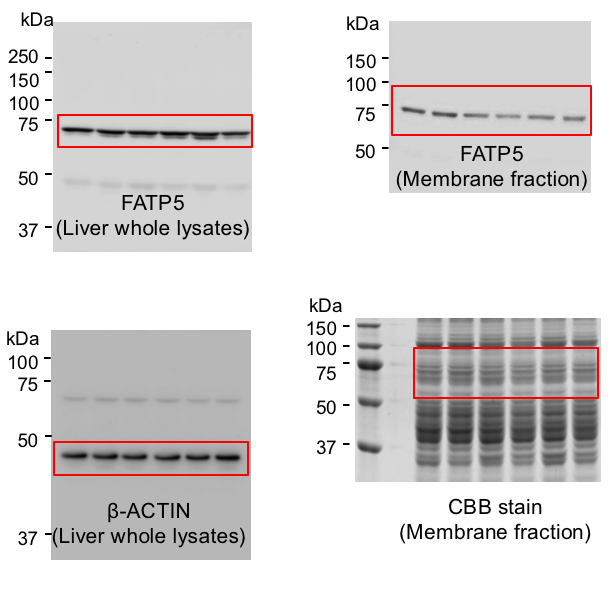


**Supplementary Figure 17** Unedited full blots of Figure 5a and 5b


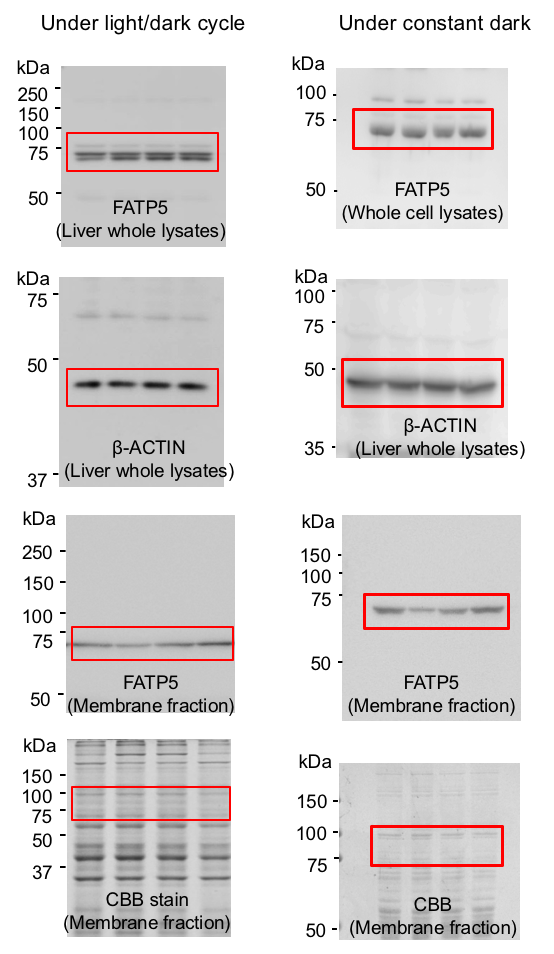


**Supplementary Figure 18** Unedited full blots of Figure 5c and d


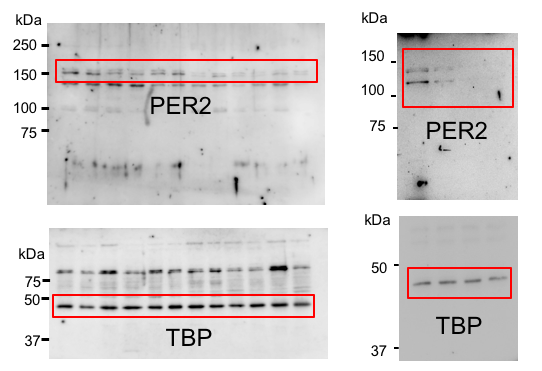


**Supplementary Figure 19** Unedited full blots of Supplementary Figure 2b


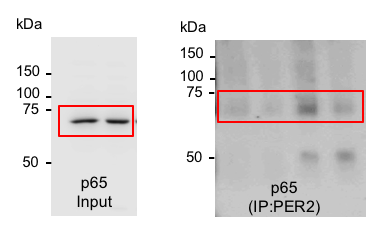


**Supplementary Figure 20** Unedited full blots of Supplementary Figure 3


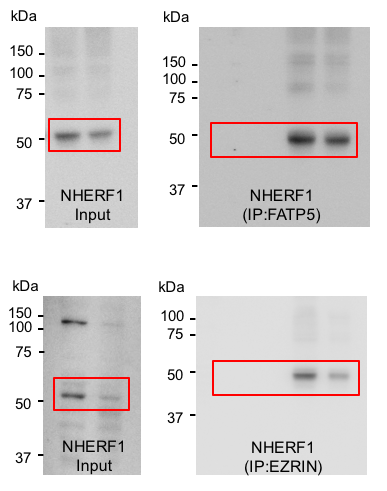


**Supplementary Figure 21** Unedited full blots of Supplementary Figure 5


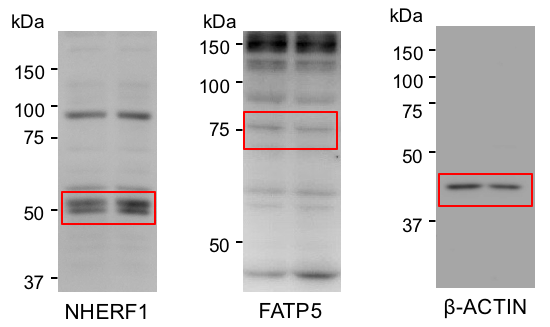


**Supplementary Figure 22** Unedited full blots of Supplementary Figure 6


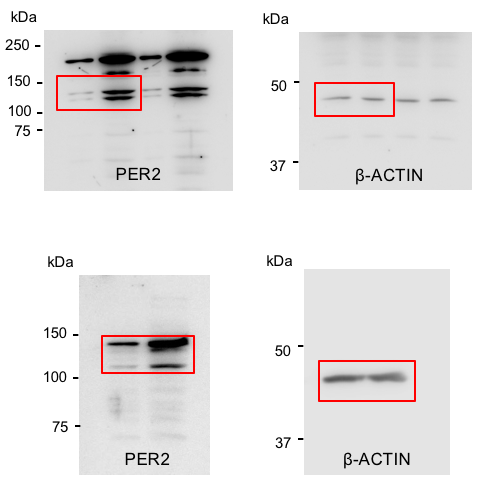


**Supplementary Figure 23** Unedited full blots of Supplementary Figure 8


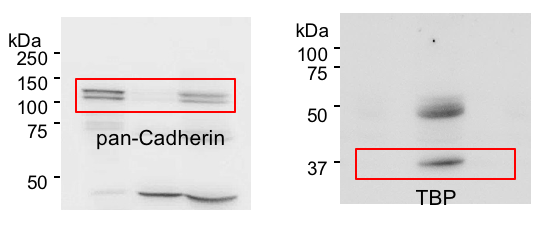


**Supplementary Figure 24** Unedited full blots of Supplementary Figure 9

**Supplementary Table 1 Oligonucleotide sequences for constructing miRNA expression vectors**

| anti lacZ miRNA  (Control) | 5’-TGCTGAAATCGCTGATTTGTGTAGTCGTTTTGGCCACTGACTGACGACTACACATCAGCGATTT-3’  5’-CCTGAAATCGCTGATGTGTAGTCGTCAGTCAGTGGCCAAAACGACTACACAAATCAGCGATTTC-3’ |
| --- | --- |
| anti PER2 miRNA | 5’-TGCTGAGCTTTGGCAGACTGCTCACTGTTTTGGCCACTGACTGACAGTGAGCACTGCCAAAGCT-3’  5’-CCTGAGCTTTGGCAGTGCTCACTGTCAGTCAGTGGCCAAAACAGTGAGCAGTCTGCCAAAGCTC-3’ |
